# Olfactory Influences on Visual Categorization: Behavioral and ERP Evidence

**DOI:** 10.1093/cercor/bhaa050

**Published:** 2020-03-30

**Authors:** Thomas Hörberg, Maria Larsson, Ingrid Ekström, Camilla Sandöy, Peter Lundén, Jonas K Olofsson

**Affiliations:** 1 Gösta Ekman Laboratory, Department of Psychology, Stockholm University, Sweden; 2 Department of Linguistics, Stockholm University, Sweden; 3 Aging Research Center (ARC), Karolinska Institute, Sweden

**Keywords:** event-related brain potentials, object categorization, olfaction, olfactory–visual processing, visual dominance

## Abstract

Visual stimuli often dominate nonvisual stimuli during multisensory perception. Evidence suggests higher cognitive processes prioritize visual over nonvisual stimuli during divided attention. Visual stimuli should thus be disproportionally distracting when processing incongruent cross-sensory stimulus pairs. We tested this assumption by comparing visual processing with olfaction, a “primitive” sensory channel that detects potentially hazardous chemicals by alerting attention. Behavioral and event-related brain potentials (ERPs) were assessed in a bimodal object categorization task with congruent or incongruent odor–picture pairings and a delayed auditory target that indicated whether olfactory or visual cues should be categorized. For congruent pairings, accuracy was higher for visual compared to olfactory decisions. However, for incongruent pairings, reaction times (RTs) were faster for olfactory decisions. Behavioral results suggested that incongruent odors interfered more with visual decisions, thereby providing evidence for an “olfactory dominance” effect. Categorization of incongruent pairings engendered a late “slow wave” ERP effect. Importantly, this effect had a later amplitude peak and longer latency during visual decisions, likely reflecting additional categorization effort for visual stimuli in the presence of incongruent odors. In sum, contrary to what might be inferred from theories of “visual dominance,” incongruent odors may in fact uniquely attract mental processing resources during perceptual incongruence.

## Introduction

It is commonly assumed that visual impressions have an especially important role in human perception and cognition. This notion has a long history; for example, Aristotle wrote that vision “in its direct effects, is the superior sense” (Aristotle 2010). In a similar vein, Immanuel Kant postulated vision to be the noblest of all the senses, because it “has the widest sphere of perception in space” (Kuehn 2006). A strong emphasis on visual processing is also present in the neurocognitive literature. For more than 40 years, experiments have shown that processing of visual stimuli “dominates” other senses in multisensory perception (e.g., Colavita 1974; Sinnett et al. 2007; Koppen and Spence 2007a). In a pioneering study, Colavita (1974) found that auditory stimuli often are neglected when presented concurrently with visual stimuli. This effect that has been replicated in more recent studies (e.g., Sinnett et al. 2007; Koppen and Spence 2007a). Similarly, in the “McGurk effect,” the perception of speech sounds is influenced by incongruent visual–articulatory information (McGurk and MacDonald 1976; Traunmüller and Öhrström 2007). In visual–tactile multisensory decisions, participants rely more on visual than tactile information (e.g., Welch and Warren 1980) as visual stimuli distract from concurrent tactile stimulation (Hartcher-O’Brien et al. 2010). Visual input thus appears to be favored in multisensory perception. Higher neurocognitive processes also appear to prioritize visual over nonvisual stimuli. When attention is divided between visual and auditory channels, cortical activity in terms of ERPs indicates that also response selection—a higher cognitive process only indirectly related to perceptual processing—is impaired for auditory but not for visual decisions (Falkenstein et al. 1991; Hohnsbein et al. 1991). However, some studies have also found auditory or tactile stimuli to dominate visual stimuli either under highly specific conditions (e.g., Shams et al. 2000; Ernst and Banks 2002; Repp and Penel 2002; Ngo et al. 2011; Robinson and Sloutsky 2013), in children (e.g., Robinson and Sloutsky 2004) or in specific subpopulations (Li et al. 2019).

On the one hand, the “visual dominance” effects have been suggested to stem from a sensory-level advantage of visual input over other sensory modalities, either due to the visual sensory channel having priority over other sensory channels (e.g., Colavita 1974; Colavita et al. 1976; Colavita and Weisberg 1979) or because of greater attention-capturing qualities or greater salience of visual stimuli in comparison to stimuli in other modalities (e.g., Koppen and Spence 2007a). Others have, on the other hand, suggested that visual dominance results from an attentional bias towards visual input (e.g., Posner et al. 1976; Sinnett et al. 2007). Posner et al. (1976) suggested that in order to compensate for the fact that visual stimuli are less alerting than other stimuli, selective attention is by default directed towards visual stimuli, resulting in less attentional resources to other sensory modalities. More recently, visual dominance has been suggested to be a consequence of the visual system actively inhibiting nonvisual processes (Spence et al. 2012).

Olfaction provides an interesting test of the visual dominance framework. Although less well-investigated than “higher” senses, olfaction has been suggested to mediate powerful alerting cues to attract attention and enable processing in other senses (Herrick 1933). This alerting capacity has more recently been theorized as the defining feature of olfaction. In particular, the olfactory system might be particularly sensitive to contextually inappropriate or novel odors, which signal potentially hazardous chemicals in the environment (Köster et al. 2014). Incongruent odor cues might therefore attract attention and thereby provide disproportional influence over vision (i.e., dominance). While the awareness of an ambient odor can be effectively eliminated by engaging in a demanding visual perception task (Forster and Spence 2018), no previous study has directly compared how olfaction and vision compete for processing resources under conditions of equal task relevance. Prior research shows that congruent visual information facilitates olfactory-based detection (Gottfried and Dolan 2003; Olofsson et al. 2013, discrimination (Dematte et al. 2008) and identification (Olofsson et al. 2013; Höchenberger et al. 2015) but congruent olfactory information similarly facilitates visual-based identification (Seigneuric et al. 2010; Zhou et al. 2010) and motion perception (Kuang and Zhang 2015).

**Figure 1:**
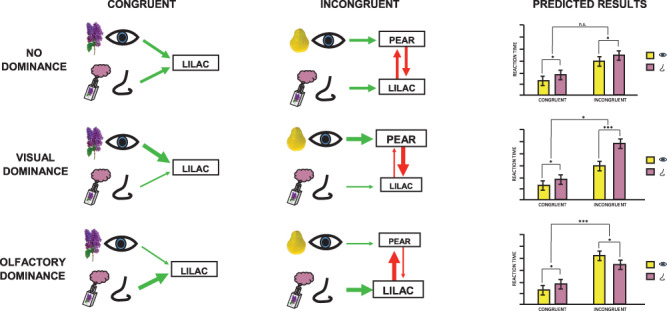
Illustration of how sensory dominance is conceptualized. Left panel: Congruent visual–olfactory stimuli (e.g., lilac picture and lilac odor) lead to a bimodal activation of the same lilac representation under assumptions of no dominance, visual dominance, or olfactory dominance. Middle panel: Incongruent visual–olfactory stimuli (e.g., pear picture and lilac odor) lead to a competitive activation, resulting in between-modality inhibition of the two representations. Under no dominance, inhibition strength is symmetric, resulting in an equal interference effect on behavioral results. Under visual dominance, the olfactory representation is to a greater extent inhibited by the visual representation, resulting in delayed olfactory decisions. Under olfactory dominance, on the other hand, the olfactory representation has a stronger inhibitory effect on the visual representation, resulting in delayed visual decisions. Right panel: Hypothesized response times corresponding to each dominance assumption.

In this preregistered study (Hörberg and Olofsson 2018, osf.io/7qnwu/), we tested the generality of visual dominance by assessing task interference across olfactory–visual sensory channels. We posited that perceptual dominance would involve a pattern of “asymmetric” activation and inhibition between sensory systems due to the salience or alerting effects of the dominant sensory input (Spence et al. 2012). We further assumed that perceptual dominance is modulated by selective attention in that successful processing in the dominated modality would require additional resources to overcome interference from dominant cues (e.g., Posner et al. 1976; Sinnett et al. 2007; Koppen and Spence 2007a). We devised a bimodal odor–picture categorization task with a delayed auditory target. Participants categorized familiar objects (lemon, pear, lavender, lilac) as fruits or flowers. The task was designed to be rapidly and accurately performed based on either olfactory or visual cues, following previous work (Olofsson et al. 2012, 2014). On each trial, an odor and a picture were simultaneously presented. After a short delay, these cues were followed by a tone that indicated whether the fruit/flower decision should be based on the odor or the picture. That is, in order to provide ample time for sensory processing of both odors and pictures (Olofsson et al. 2014, 2018), the tone needed to make a response decision was delayed. In order to engage competition for processing resources, the odor–picture pair was incongruent on half of the trials (e.g., lemon odor, lavender picture). We assumed that when visual and olfactory stimuli activate two conflicting representations, the dominant sensory system will be less vulnerable to such cross-modal distraction and retain a relatively high behavioral performance. Incongruent stimuli in the dominant sensory modality should interfere more with the nondominant modality than vice versa, resulting in a relative behavioral advantage during the incongruent condition for decisions in the dominant modality (see Fig. 1).

Although response decisions were linked to a delayed, auditory stimulus, we hypothesized that visual-based decisions should be overall faster and/or more accurate than olfactory-based decisions. Prior results have shown that such odor decisions triggered by auditory targets involve a reactivation of olfactory-specific cortical pathways in the orbitofrontal and the anteromedial temporal cortex and lead to less rapid and accurate responses compared to visual decisions (Olofsson et al. 2014). In Figure 1, which shows hypothetical outcomes, this general response advantage for visual stimuli over olfactory stimuli would be observed as a main effect of modality (upper panel). Based on the vast literature on visual dominance, we further assumed that if visual processing dominates olfactory processing, incongruent visual stimuli should interfere more with olfactory categorization than incongruent olfactory stimuli would interfere with visual categorization (middle panel).

If, on the other hand, olfactory processing dominates visual processing, incongruent olfactory stimuli should interfere more with visual categorization than vice versa (lower panel). In other words, a perceptual dominance account would predict an interaction effect between congruence and modality, and this would be observed in either reaction times (as illustrated with hypothetical bar plots in Fig. 1, right panel) or in response accuracies. Congruent cues served as our control condition.

We also investigated cortical responses time-locked to the presentation of the auditory target, using ERPs. This would allow us to draw further conclusions regarding the cortical processing sequence underlying perceptual dominance. We were interested in ERP effects that would correspond to behavioral effects and thereby aid interpretation. Further, we explored the notion that perceptual dominance involves asymmetric inhibition between perceptual systems. We hypothesized that if perceptual dominance is a consequence of asymmetric inhibition, successful categorization of stimuli in the dominated sensory channel should require additional attentional resources in order to compensate for the strong interference effect of the incongruent input in the dominating channel.^1^ The allocation of attentional resources during stimulus categorization has been linked to the P300 ERP response, a positive, centro-parietal or centro-frontal wave around 300-ms poststimulus onset (e.g., Kutas et al. 1977; Picton 1992; Sutton et al. 1965; Verleger 1997; and Polich 2011 for a review). Stimulus categorization involves the integration of external stimuli with working memory representations, a process that is mediated by attention (Kok 2001). It has been suggested that P300 amplitude reflects the attentional demands of the categorization task (Kok 2001), which, in turn, co-occurs with inhibition of task-unrelated activity (Polich 2007). In the context of the present study, categorizing stimuli during cross-modal interference can therefore be expected to result in an enhanced P300 wave. Further, more attentional resources might be required to inhibit the sensory representation in the dominant sensory system. This should result in a more pronounced P300 wave during categorization in the nondominant sensory channel.

In addition to the ERP response to the auditory target, we also analyzed ERPs time-locked to bimodal cue presentation, thereby investigating the cortical response to congruent versus incongruent stimulus processing (i.e., the N400 response, see, e.g., Mudrik et al. 2010). However, as these analyses are not crucial for our research questions, they are presented in the Supplementary Materials.

## Materials and Methods

### Participants

Participants were recruited at Stockholm University via online advertisements. Participation was compensated with either gift vouchers worth 200 SEK or course credit. Power analyses based on behavioral and ERP pilot data and simulations (see Supplementary Materials) showed that a sample size of 40 individuals would be sufficient (power > 0.8) even for small effect sizes (standardized βs = 0.2). The initial sample therefore consisted of 46 healthy adults who reported to have normal to corrected-to-normal vision and a normal sense of smell, and who were screened for their ability to correctly identify the four stimulus odors. We excluded data from 10 participants who had less than 75% correct trials in either the congruent or incongruent conditions and data from 1 participant with missing background data. In the analyses of the behavioral data, the effective sample size therefore consisted of 35 individuals (*M* age, 31.3 years; range, 19–59 years, 16 females). In the ERP data analyses, data from an additional five participants was also excluded due to EEG artifacts, resulting in a sample size of 30 individuals (*M* age, 32.3 years; range, 19–59 years, 14 females). In order to preserve statistical power, we chose to keep these five participants for behavioral analyses. However, the results of these analyses also held with these participants excluded. All participants gave written informed consent in accordance with the Declaration of Helsinki. The study was approved by the regional ethics board 2014/2129-31/2.

### Stimuli

The stimuli consisted of four visual and four olfactory objects belonging to the categories *fruit* (lemon and pear) and *flower* (lavender and lilac). Odors were presented with an olfactometer that was controlled by the stimulus computer. We used 1–3 ml of odor essences and oils from Stockholms Aeter and Essencefabrik AB (pear “päronessens” and lilac “syrenolja”) and Aroma Creative (lemon “citron” and lavender “lavendel”).^2^ The odor identification and rating tasks indicated that the odors were easy to identify and perceived as similar in intensity, pleasantness, and specificity but differed in edibility (see Supplementary Materials). The pictures were presented on a computer screen and consisted of photographed images that were matched in size, brightness, and hue. All pictures were 10.5 cm high (subtending 6.01° vertical visual angle at a 1-m distance). The lavender and lilac pictures were 5.5 cm wide (3.15° horizontal visual angle), and the lemon and peak pictures were 8 cm wide (4.58° horizontal visual angle). The auditory targets consisted of two 0.5-s-long sinus tone that were presented in earphones. Tone amplitudes were adjusted for tone loudness. The low tone consisted of a 630-Hz tone with 60.8 dB and the high tone of 1250 Hz tone with 62.2 dB.

### Procedure

The experiment was conducted in brightly lit and well-ventilated olfactory testing room at the Gösta Ekman Laboratory, Department of Psychology, at Stockholm University. Participants were informed about the experiment and that they could abort it at any time. They were seated at a 1-m distance from the stimulus computer screen. Participants performed a training protocol in which they identified the experimental stimuli. Participants also performed perceptual odor ratings (see Supplementary Materials for details).

**Figure 2:**
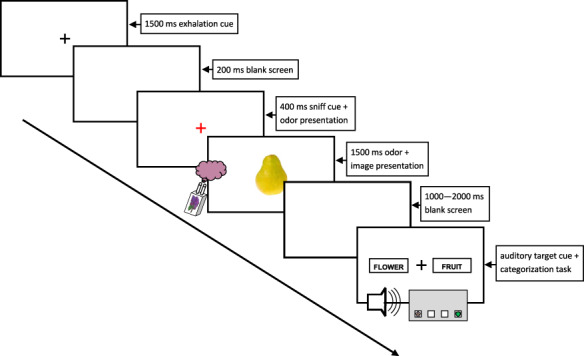
Trial structure of the categorization task. During training trials (but not in experimental trials), the object modality to be categorized was also displayed on the final screen, written above the fixation cross, in order to learn the meaning of the two target tones.

In the main experimental task, participants categorized visual or olfactory stimuli as fruit or flower. In order to investigate modality dominance, we used a categorization task with cross-modal interference. Visual and olfactory stimuli were presented concurrently in order to achieve a simultaneous bimodal percept (see below for details). On congruent trials, the same visual and olfactory objects were used (e.g., the picture and odor of pear), yielding a total of four different odor–picture pairings. On incongruent trials, objects from each of the two categories were used (e.g., the picture of pear and the odor of lilac), resulting in eight different odor–picture pairings (see, e.g., Olofsson et al. 2012, 2014, for a similar protocol). Importantly, in order to remove bias due to processing speed differences between visual and olfactory perception, the auditory target cues that informed about the object target (i.e., picture or odor) were delayed by a varying interval of 1000–2000-ms poststimulus offset. This allowed for statistical analyses of the possible effect of lag time. Further, the cue onset timing and the delayed auditory target minimized the risk that sensory processing speed would influence the results (see Supplementary Materials). A categorization trial is illustrated in Figure 2.

Each trial begun with the presentation of the odor. First, a black fixation cross appeared for 1500 ms in the center of the screen. It indicated that it was time to exhale and prepare to sniff. Following a 200-ms blank screen, a sniff cue (red fixation cross) appeared. The odor was simultaneously released by the olfactometer. At 400 ms after olfactory stimulation (and sniff cue) onset, the picture appeared in the center of the screen. It was presented together with the odor for 1500 ms. In other words, pictures were presented with a lag of 400 ms relative to the olfactometer trigger. This was done in order to compensate for the processing time difference between visual and olfactory stimuli. Whereas visual detection RTs are on average about 300 ms (e.g., Collins and Long 1996; Amano et al. 2006), olfactory detection RTs are around 800 ms when following the current protocol (Olofsson et al. 2013, 2014). Also visual ERPs occur about 300–400 ms before olfactory ERPs (Geisler and Polich 1994; Alexander et al. 1995; Pause et al. 1996; Romero and Polich 1996). Taken together, these findings are highly suggestive of a 350–500-ms delay in olfactory processing times and prompted our 400-ms odor–picture lag time. However, as our dependent measures (ERPs and RTs) were linked to the onset of a delayed auditory target stimuli, the exact timing of the cue onset should not be critical.

After stimulus presentation, the screen turned blank for 1000–2000 ms. Following this delay, participants were presented with the target cues (i.e., low/high sinus tone, presented together with a black fixation cross at the center of the screen) and performed the categorization task. The fixation cross was flanked by two text boxes that reminded about the button assignment (i.e., whether the left button was used for fruits and the right for flowers or vice versa). The position of the boxes (left vs. right) corresponded to the button assignment. The task was performed by pressing either the leftmost or the rightmost button of a four-button response box. Participants were encouraged to respond as quickly and accurately as possible. The four possible combinations of tone and button assignment were counterbalanced across participants. Each trial ended with a delay (minimum 1000 ms) that ensured that at least 10 s had passed since the start of the trial.

Participants conducted 128 trials, 2 (congruence) × 2 (modality) × 2 (category) × 2 (stimuli) × 8 (repetitions), evenly distributed across 4 blocks. In the incongruent trials, the target stimulus object (i.e., the sensory object to be categorized) co-occurred with either of the two incongruent stimuli equally often (e.g., pear odor co-occurred with lilac and lavender pictures on an equal number of trials). Trial presentation order within a block was randomized. At the beginning of each block, a visual display informed that the next block was about to start. The block started with a button press. In order to get familiarized with the task, participants performed a training session consisting of 16 trials, 2 (congruence) × 2 (modality) × 2 (category) × 2 (stimuli), before the actual experiment started. In the training trials, but not the experimental trials, the sensory modality to categorize was displayed on the screen (directly above the fixation cross, see Fig. 2), in order for the participants to learn the meaning of the target tones. Participants were encouraged to take short breaks in between blocks. They were told to avoid blinking and moving from the time the sniff cue was presented to the time of their response.

### Apparatus

Odors were presented birhinally with a custom-built, continuous-flow olfactometer described in detail in Lundström et al. (2010). The olfactometer was controlled using experimental PsychoPy software through a parallel port. In order to evaluate the timing of odor presentation in our experimental setup, we performed measurements of the temporal performance of the olfactometer (presented in detail in the Supplementary Materials). These showed that the onset of the odor output occurs approximately 54 ± 7 ms after the presentation of the visual sniff cue. The olfactometer has also been shown to emit a stable odor concentration over time (approximately a 0.5% decrease over a 10-min period) and to be suitable for recording olfactory ERPs (Lundström et al. 2010). The continuous airflow was set to 0.5 l/m and individual channel airflows to 2.5 l/m.

Visual stimuli were presented on a 24″ Benq XL2430-B TN-screen with 100-Hz refresh rate and a resolution of 1920 × 1080 pixels. The experiment was run on a Windows 7 PC. In the odor identification and rating tasks, participants responded with the mouse. In the categorization task, they responded with a Cedrus RB-740 Response Box (Cedrus Corporation).

### EEG Recording

EEG was recorded with a 64-pin electrode Active Two Biosemi system (Biosemi), using EEG caps (Electro-Cap International). In addition to the 64 10–20 electrodes, the Biosemi system uses an internal reference electrode (CMS), positioned in between PO3 and POz, and a ground electrode (DRL) positioned in between POz and PO4. EOG was recorded with two flat electrodes attached with an adhesive disk, one positioned at the outer canthus of the right eye and the other directly below the right eye. Data was sampled at 2048 Hz with a hardware low-pass filter at 410 Hz but down sampled to 512 Hz offline.

### Data Analysis

#### EEG Preprocessing

We performed all offline EEG preprocessing in EEGLAB (Delorme and Makeig 2004) in MATLAB (MathWorks, Inc.). The raw EEG data was down sampled to 512 Hz and band-pass filtered between 0.2 and 40 Hz, using a FIR filter with a cutoff frequency of 0.1 Hz. Irrelevant parts of the filtered data were then removed to select experimental trial segments ranging from 1000 ms prior to the start of the trial (i.e., the presentation of the black fixation cross) to 1000 ms after the response, with training session trials included. Channels were defined as bad if the amplitude difference exceeded 500 mV in more than 50% of 1000-ms time windows, if their correlation with their robust estimates as calculated from the signal of the 16 neighboring channels was less than 0.75, or if their signal-to-noise ratio deviated with more than 4 standard deviations (SDs) from the channel mean signal-to-noise ratio of all channels. On average, five channels in each participant data set was bad (min: 0, max: 27). Bad channels were interpolated using spherical splines, and the data was re-referenced to the average of all channels, using a full-rank average.^3^ We then performed ocular artifact rejection using independent components analysis (ICA). The data used for ICA decomposition was high-pass filtered at 1 Hz, trimmed of noisy data by visual inspection, and analyzed with the AMICA EEGLAB plugin. The resulting ICA components were transferred back to the original 0.2–40.0-Hz band-pass-filtered data. Ocular artifact ICA components were automatically identified and removed using the icablinkmetrics plugin (Pontifex et al. 2017), as based on their relationships with activity in the vertical EOG channel, the horizontal EOG channel, or the mean of channels Fp1, AF7, FCz, Fp2, and AF8. We used a correlation coefficient threshold of 0.9, a convolution coefficient threshold of 0.1, and an artifact reduction threshold of 10% that had to be statistically significant at the .001 alpha level. On average, three components were identified as artifactual in each participant data set (min: 0, max: 6).

The artifact-corrected data from the experimental trials were again re-referenced to the full-rank average of all channels and then divided into −200 to 1000-ms epochs relative to onset of the visual stimuli and onset of the auditory target. Epochs were baseline-corrected by subtracting the mean of the 200-ms prestimulus period. Epochs were removed if they had a ± 120-uV amplitude difference in any channel, if the amplitude difference in any channel deviated by more than 6 SDs from the mean amplitude channel difference in all epochs, or if the amplitude difference of four channels deviated by more than 4 SDs from their mean channel amplitude differences in all epochs. We removed 16% of all epochs using these criteria (per-participant minimum, 5%; maximum, 58%). We also excluded epochs from trials with RTs below 200 ms or above 5000 ms (similar to Olofsson et al. 2012). Five participants with less than 15 epochs remaining in any condition were excluded from subsequent EEG data analyses.

#### Statistical Analyses

All results were analyzed in the statistical software R (R Core Development Team 2018), using custom-made analysis scripts (available at osf.io/7qnwu/). As stated in the preregistration, we performed Bayesian mixed-effect modeling in the Stan modeling language (Stan Development Team 2017), using the R package Rstan (Stan Development Team 2018). Response times and ERP amplitudes were analyzed with linear mixed-effect modeling and accuracy with logistic mixed-effect modeling. Full model specifications and model priors are presented in the Supplementary Materials. Inferences about parameter effects (e.g., the congruence × modality interaction) were done on the basis of the parameter credibility intervals (CIs). We considered a parameter 95% CI not including zero as evidence for an effect of the parameter at hand. We also report the posterior probability (*P*) of a parameter being zero or taking on values in the opposite direction of the mean parameter estimate, multiplied by 2. All Bayesian analyses were complemented with frequentist mixed-effect modeling (see Supplementary Materials). All models contained fixed effects for the independent variables congruence (congruent vs. incongruent) and modality (visual vs. olfactory), and for the congruence × modality interaction. The models also included fixed effects for the following potential confounders.

*Trial number*. In order to control for any learning effects remaining after the initial training session, we included trial number as a control variable.

*Delay*. The delay between olfactory–visual cues and auditory targets varied randomly between 1000 and 2000 ms, in steps of 200 ms. A longer delay gives participants more time for stimulus processing and response preparation and might thus result in shorter response times and higher accuracies. We also used this varying delay as a control variable, to test whether longer delay times would be more beneficial for any particular sensory system.

*Object category*. The object category, fruit or flower, was also included in order to control for any potential differences in categorization.

*Gender.* The gender of the participant was also included as some studies have found women to have somewhat better olfactory perceptual abilities than men (e.g., see Doty and Cameron 2009 for a review).

*Similarity index.* In order to control for a potential influence of between-modality differences in perceived stimulus similarity, we also included a between-category similarity index as a control variable. This index aimed to capture the participant-specific between-category similarity, that is, the individually perceived similarity between a cue category stimulus (e.g., lemon of the fruit category) and the two stimuli of the other, competing category (i.e., lilac and lavender of the flower category). We wanted to quantify whether a high between-category similarity could render the categorization task more difficult, as the cue stimulus should be harder to differentiate from the stimuli of the competitor category. Within-category similarity, on the other hand, should not influence categorization, since a confound (e.g., confounding pear and lemon) would not affect the categorical decision (fruit). This index was calculated on the basis of between-category similarity ratings of the stimuli (see Supplementary Materials). First, in order to make similarity ratings comparable across participants, ratings were standardized within participants, ensuring that participant rating means and SDs were the same for each participant. The between-category similarity index was then calculated within each participant, cue stimulus, and modality as the mean of the standardized similarity ratings involving the stimulus at hand. Since participants rated the stimuli for their similarity to each of the two competitor category stimuli twice (e.g., two similarity ratings of lemon–lilac and two of lemon–lavender), this index was the mean of four ratings.

All models also include random intercepts for participants and items, the latter differentiating between all possible visual and olfactory stimulus combinations. Thereby, we control for any systematic differences between subject and stimulus pairs. We also included a by-participant random slope for trial number, thereby controlling for any differences in learning between participants. RT data was log-transformed in order to ensure normality. All continuous covariates were standardized by subtracting the mean and, following Gelman and Hill (2006), divided by 2 SDs. Categorical variables were effect-coded through centering.^4^ The main effects are therefore tested against the grand mean of the data. In the event of congruence × modality interaction effects, we conducted simple effect follow-up analyses, testing the effect of modality in the congruent and the incongruent conditions separately. This was done by including three dummy-coded predictors either for visual-congruent, olfactory-congruent, and visual-incongruent (testing modality within incongruent trials), or for visual-incongruent, olfactory-incongruent, and visual-incongruent (testing modality within congruent trials).

#### ERP Analyses

We investigated corresponding ERP effects time-locked to the auditory target that were related to the increased processing demands of incongruent compared to congruent trials and their observed interactions with sensory modality. However, since this is the first study of its kind to include ERP data, we did not make any specific predictions in terms of exact time windows and regions of interest (ROIs). These were chosen on the basis of previous literature and visual inspection of the data. In order to further confirm our choice of time windows, we also performed cluster-based permutation analysis (Maris 2012; similar too, e.g., Maris and Oostenveld 2007), using custom-made analysis scripts (available at osf.io/7qnwu/). Although this method does not provide evidence for whether an ERP effect occurs in a particular spatiotemporal region (i.e., a specific cluster), it allows for the identification of regions of interest for further investigation and provides evidence for a difference in the ERP response to two conditions more generally (i.e., by rejecting the null hypothesis that the ERP data of both conditions come from the same probability distribution, see Maris and Oostenveld 2007; Sassenhagen and Draschkow 2019). Importantly, cluster-based permutation does not suffer from problems of making multiple comparisons, resulting in an inflation of the risk of falsely rejecting the null hypothesis (e.g., Benjamini and Hochberg 1995; Dunnett 1955; Hochberg 1988). Our implementation of the cluster-based permutation test is highly similar to that of the EEG analysis tool FieldTrip (Oostenveld et al. 2011). First, *t*-values for the ERP condition differences at each spatiotemporal location are calculated. *t*-values above 2 or below −2 from neighboring spatiotemporal locations are then grouped into positive and negative clusters and summed for each cluster. Two probability distributions of *t*-values is then calculated on the basis of cluster-based Monte Carlo permutation. This involves randomly assigning data sets to conditions multiple times and, for each permutation, calculating cluster-based, summed *t*-values, in the same way that was done in the original data. The distributions of maximum and minimum *t*-values from each permutation then serve as the probability distributions against which the observed summed *t*-values are tested. This distribution approximates the probability distribution for the largest *t*-values that can be expected under the null hypothesis that the ERP data from both conditions come from the same probability distribution and therefore do not differ. In our analyses, we included clusters with *t*-values that had at most a 5% probability to be observed under the null hypothesis (i.e., α = <0.05). For stimulus presentation ERP data, we performed a cluster-based permutation test that compared the congruent and incongruent condition (reported in the Supplementary Materials). For auditory target ERPs, we first compared the congruent and incongruent conditions across modalities and then compared modality differences within the congruent and the incongruent conditions separately.

We also performed analyses on ERP data on single-trial (rather than subject average) mean ERP amplitudes across time windows and electrode groups (similar to Frömer et al. 2018), using Bayesian and frequentist linear mixed-effect models. These analyses were conducted on ERPs time-locked to the stimulus presentation (reported in the Supplementary Materials), on the one hand, and to the auditory target, on the other. As motivated by the results of our cluster-based permutation analyses (see below), we performed analyses on mean amplitudes in the P300 time window, ranging from 320 to 580 ms, across the centro-frontal (CF) scalp region which consisted of electrodes AF3, Afz, AF4, F1, Fz, F2, FC1, FCz, and FC2. We also conducted three separate analyses in the late 600–700-, 700–800-, and 800–900-ms time windows across the centro-occipital (CO) region, consisting of P4, P2, Pz, P1, P3, PO8, PO4, POz, PO3, PO7, O2, Oz, and O1 (see Supplementary Fig. 8 in Supplementary Materials), where a positive slow wave (PSW) effect was observed.

As a final step, we also investigated the relationship between RTs and single-trial mean ERP amplitudes in the P300 and the late PSW time windows, again using both Bayesian and frequentist linear mixed-effect models. In addition to the fixed effects for the confounders listed above, these models contained dummy-coded predictors for each condition (olfactory-congruent, visual-congruent, olfactory-incongruent, and visual-incongruent) and corresponding RT interaction terms (e.g., RT × olfactory-congruent). Intercept terms were excluded. Each interaction term therefore tests whether the RT slope within each condition differs from zero, that is, whether within-condition ERP amplitudes are negatively or positively correlated with RTs.

## Results

In the following, we first report results of behavioral data and then results of ERP data.

### Behavioral Data

#### Reaction Times

**Figure 3:**
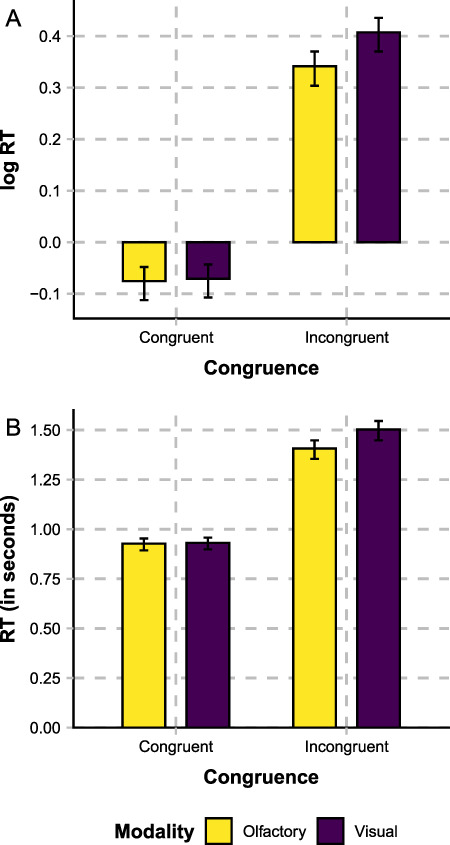
Log RTs (panel A) and RTs in seconds (panel B), differentiated on the basis of congruence (congruent vs. incongruent) and the modality of the target stimulus object (olfactory vs. visual). Error bars show 95% CIs as estimated by the Bayesian model.

Mean RTs from each condition (i.e., olfactory-congruent, visual-congruent, olfactory-incongruent, and visual-incongruent) are shown in Figure 3. The results of the Bayesian linear mixed-effect models of log RTs are shown in Table 1.

**Table 1:** Results of the Bayesian linear mixed-effect models of log RT data

Parameter	Beta	SE	CI lower	CI upper	*P*
Intercept	0.17	0.07	0.04	0.30	0.009
Congruence	0.45	0.03	0.38	0.52	<0.0001
Modality	0.04	0.02	0.00	0.08	0.041
Congruence × modality	0.06	0.03	0.00	0.11	0.041
Congruent: modality	0.01	0.03	−0.04	0.06	0.571
Incongruent: modality	0.07	0.03	0.02	0.12	0.009
Category	0.02	0.02	−0.02	0.05	0.383
Sex	0.14	0.13	−0.11	0.40	0.280
Trial	−0.35	0.03	−0.42	−0.28	<0.0001
Delay	0.02	0.01	−0.01	0.04	0.229
Similarity	0.01	0.02	−0.03	0.05	0.516

*P* values are the percentage of parameter samples being zero or taking on values in the opposite direction of the mean parameter estimate, out of a 4000-sample parameter posterior distribution.

As expected, categorization RTs are faster for congruent than for incongruent stimuli, as confirmed by the fact that the CI of congruence does not include zero. Further, RTs for visual-based responses are “slower” than olfactory-based responses, when the stimuli are incongruent. Although there is a main effect of modality and therefore a general RT difference between modalities, there is also a congruence × modality interaction effect. Bayesian follow-up simple effect analyses did not find a modality difference within congruent trials but instead within the incongruent trials. Overall then, RTs do not differ between olfactory and visual categorizations when the cues are congruent. When the cues are incongruent, on the other hand, RTs are on average 70 ms faster for olfactory than for visual categorizations, indicating a higher degree of olfactory interference with visual categorization than vice versa.

#### Accuracy

**Figure 4:**
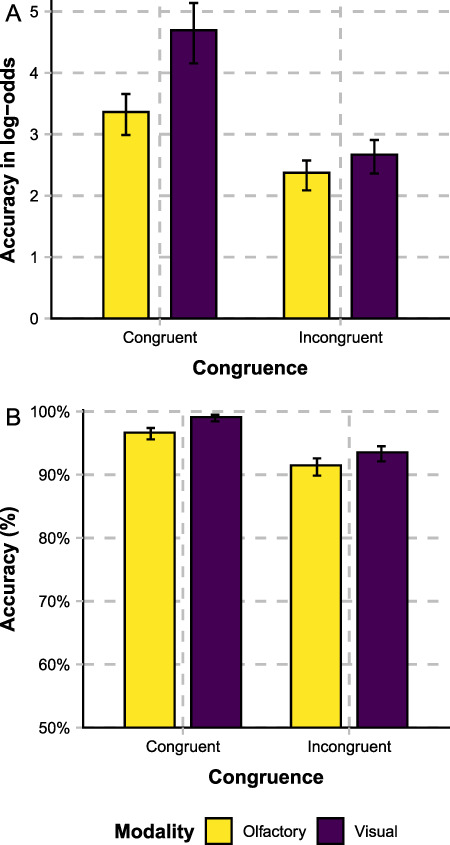
Accuracy in log-odds (panel A) and percent (panel B), differentiated on the basis of congruence (congruent vs. incongruent) and the modality of the target stimulus object (olfactory vs. visual). Error bars show 95% CIs as estimated by the Bayesian model.

Accuracy of each condition is shown in Figure 4. The results of the Bayesian logistic mixed-effect model on the accuracy data is shown in Table 2.

**Table 2:** Results of the Bayesian logistic mixed-effect models of accuracy data

Parameter	Beta	SE	CI lower	CI upper	*P*
Intercept	3.68	0.26	3.18	4.18	<0.0001
Congruence	−1.34	0.33	−1.96	−0.63	0.001
Modality	0.58	0.24	0.11	1.07	0.012
Congruence × modality	−0.8	0.35	−1.49	−0.11	0.023
Congruent: modality	1.13	0.36	0.44	1.83	0.001
Incongruent: modality	0.12	0.24	−0.35	0.59	0.627
Category	0.19	0.17	−0.13	0.52	0.252
Sex	−0.31	0.37	−1.07	0.46	0.391
Trial	0.54	0.24	0.07	1.04	0.020
Delay	−0.12	0.15	−0.4	0.18	0.426
Similarity	−0.26	0.21	−0.68	0.17	0.210

*P* values are the percentage of parameter samples being zero or taking on values in the opposite direction of the mean parameter estimate, out of a 4000-sample parameter posterior distribution.

As expected, accuracy is higher for congruent than for incongruent stimuli, as shown by the congruence main effect. Further, accuracy is higher for visual targets when the stimuli are congruent, but do not differ between modalities when the stimuli are incongruent. There is a modality main effect and therefore a general difference in accuracy between modalities. However, there is also a congruence × modality interaction effect. Follow-up simple effect analyses showed that accuracy is higher for visual targets in the congruent trials but that there is no accuracy difference between modalities in the incongruent trials.

Taken together, although visual stimuli are categorized more accurately when the stimuli are congruent, there is no categorization advantage for visual stimuli in the incongruent conditions, neither in terms of RTs nor in terms of accuracy. That is, our behavioral data do not provide any evidence for overall visual dominance when incongruent odors are presented. Instead, incongruent odors seem to interfere more with visual decisions than vice versa, as indicated by the faster categorization RTs for incongruent olfactory targets in comparison to incongruent visual targets. Contrary to the well-established notion that visual processing dominates other sensory input, these findings instead suggest olfactory dominance over visual input.

### ERP Data

#### Cluster-Based Permutation Analyses

**Figure 5:**
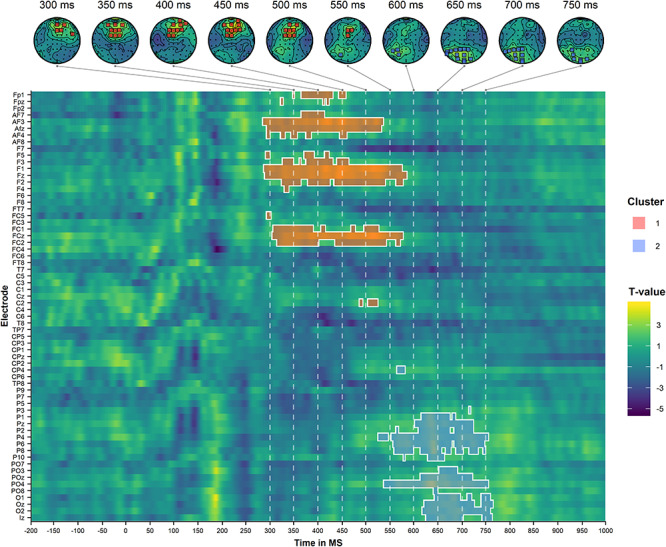
Results of the cluster-based permutation analysis of the auditory target ERPs, comparing the incongruent condition to the congruent condition (collapsed over modality). The top panel illustrates the scalp topographies of the identified clusters (ordered by size) at selected time points. The bottom panel illustrates the spatiotemporal distribution of the identified clusters. The dashed lines correspond to the selected time points of the topoplots in the upper panel.

We first conducted a cluster-based permutation analysis comparing the incongruent to the congruent condition, collapsing over modality. This analysis identified one positive cluster with a centro-frontal and centro-parietal distribution within the 300–580-ms time window and another positive cluster with a centro-parietal and centro-occipital distribution in the 550–750-ms time window. These clusters are illustrated in Figure 5.

**Figure 6:**
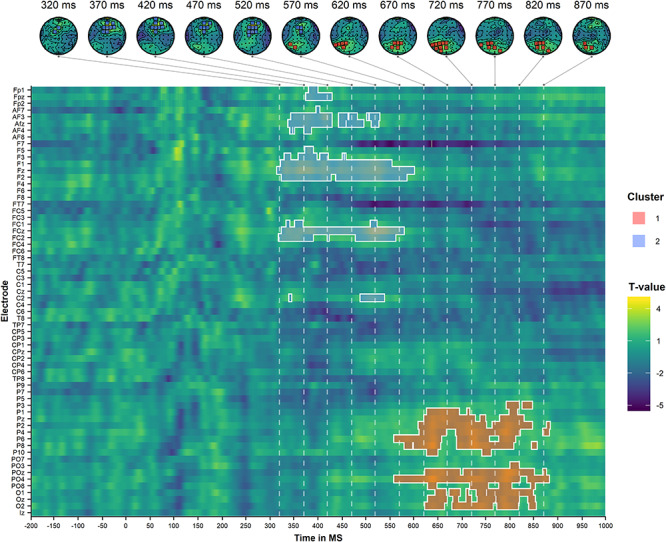
Results of the cluster-based permutation analysis of the auditory target ERPs during visual decisions, comparing the incongruent condition to the congruent condition. The top panel illustrates the scalp topographies of the identified clusters (ordered by size) at selected time points. The bottom panel illustrates the spatiotemporal distribution of the identified clusters. The dashed lines correspond to the selected time points of the topoplots in the upper panel.

We also conducted analyses within the visual and olfactory conditions separately, comparing the incongruent to congruent condition within each modality independently. Our analysis within the visual conditions identified one significant positive cluster with a centro-frontal and centro-parietal distribution in the 320–600-ms time window and another positive cluster with a centro-parietal and centro-occipital distribution in the 550–900-ms time window. These clusters are illustrated in Figure 6.

**Figure 7:**
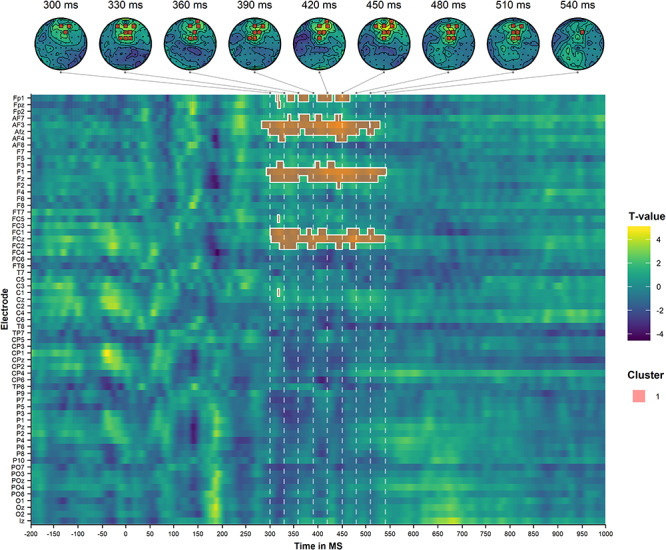
Results of the cluster-based permutation analysis of the auditory target ERPs during olfactory decisions, comparing the incongruent condition to the congruent condition. The top panel illustrates the scalp topographies of the identified clusters at selected time points. The bottom panel illustrates the spatiotemporal distribution of the identified clusters. The dashed lines correspond to the selected time points of the topoplots in the upper panel.

The analysis in the olfactory conditions identified one centro-frontally and centro-parietally distributed positive cluster in the 300–550-ms time window, illustrated in Figure 7.

These findings suggest that there is a domain-general congruence effect in the centro-frontal/centro-parietal scalp region around the 300–600-ms time region (i.e., a P300 effect). They further indicate that there also is a congruence effect in the centro-parietal/centro-occipital region around the 550–900-ms time window (i.e., a PSW effect) that, importantly, has a longer latency and is more pronounced for visual than for olfactory decision trials.

#### Mean ERP Analyses

**Figure 8:**
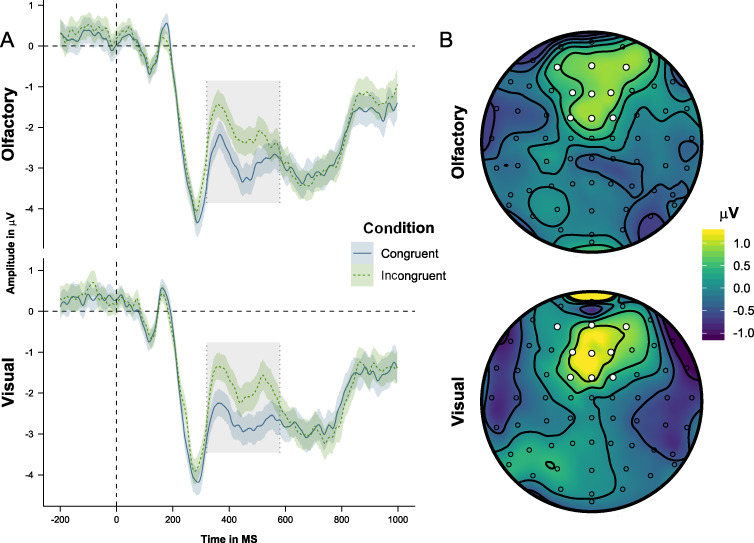
Panel A: Grand average ERPs time-locked to the presentation of the auditory targets, averaged across the CF ROI, and differentiated on the basis of congruence and modality. Shaded areas show 95% confidence intervals. Gray areas mark the 320–580-ms time window. Panel B: Topography of the incongruent–congruent grand average difference of the P300 (320–580 ms) time window.

**Figure 9:**
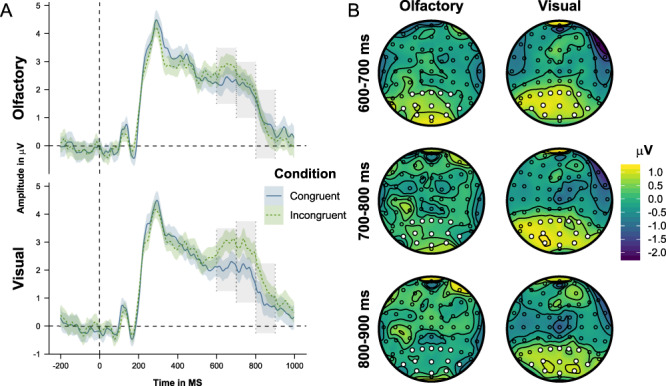
Panel A: Grand average ERPs time-locked to the presentation of the auditory targets, averaged across the CO ROI, and differentiated on the basis of congruence and modality. Shaded areas show 95% confidence intervals. Gray areas mark the 600–700-, 700–800-, and 800–900-ms time windows. Panel B: Topography of the incongruent–congruent grand average differences of the 600–700-, 700–800-, and 800–900-ms time windows.

We present grand average auditory target ERPs across the CF and CO ROIs, together with topoplots of congruent–incongruent grand average differences in the P300 (320–580 ms) and PSW (600–700, 700–800, and 800–900 ms) time windows (Figs 8 and 9). The congruence and modality effects in the Bayesian linear mixed-effect models conducted on mean ERP amplitudes are shown in Table 3. We first present analyses of the P300 effect and then analyses of the PSW effects.

**Table 3:** Congruence and modality effects in the Bayesian linear mixed effect models of mean ERP amplitudes in the P300 and PSW time windows

ERP effect	Parameter	Beta	SE	CI lower	CI upper	*P*
P300	Congruence	0.71	0.15	0.40	1.01	0.001
	Modality	0.14	0.18	−0.20	0.50	0.433
	Congruence × modality	0.13	0.23	−0.31	0.56	0.605
PSW1	Congruence	0.66	0.17	0.31	1.00	<0.0001
	Modality	0.14	0.22	−0.29	0.58	0.549
	Congruence × modality	0.22	0.29	−0.34	0.78	0.458
	Congruent: modality	−0.01	0.27	−0.54	0.53	0.949
	Incongruent: modality	0.30	0.26	−0.21	0.80	0.262
PSW2	Congruence	0.54	0.20	0.15	0.93	0.014
	Modality	0.26	0.22	−0.17	0.69	0.245
	Congruence × modality	0.55	0.27	0.03	1.07	0.039
	Congruent: modality	−0.06	0.26	−0.59	0.45	0.809
	Incongruent: modality	0.57	0.27	0.05	1.08	0.033
PSW3	Congruence	0.28	0.19	−0.10	0.63	0.137
	Modality	0.22	0.23	−0.23	0.66	0.338
	Congruence × modality	0.77	0.30	0.17	1.35	0.012
	Congruent: modality	−0.19	0.27	−0.71	0.33	0.480
	Incongruent: modality	0.60	0.27	0.07	1.13	0.032

*P* values are the percentage of parameter samples being zero or taking on values in the opposite direction of the mean parameter estimate, out of a 4000-sample parameter posterior distribution.

#### P300 (320–580 ms)

As illustrated in Figure 8, incongruent trials engender a positive response in the P300 time window in the CF ROI. The analysis of this effect showed a main effect of congruence, but no congruence × modality interaction. In other words, incongruent trials elicit a fronto-centrally distributed positivity in the P300 time window which is independent of cue modality.

#### PSW (600–900 ms)

As shown in Figure 9, incongruent trials elicit a more pronounced positive slow wave in the 600–900-ms time window in the CO ROI, an effect that is likely to reflect increased processing demands. Importantly, this effect differs between modalities in that it has a later amplitude peak (683 vs. 652 ms) and longer latency when the target stimulus object is visual compared to when it is olfactory (see Fig. 9). In order to investigate the latency difference, we conducted three separate analyses on ERP amplitude averages in each of the three consecutive 100-ms windows. In the 600–700-ms latency range, we found a main effect of congruence, but no congruence × modality interaction, similar to the P300 results. In the 700–800-ms time window, the congruence main effect remained but, importantly, was complemented by a congruence × modality interaction. In the 800–900-ms time window, finally, there was no main effect of congruence but instead a congruence × modality interaction. Follow-up simple effect analyses showed no modality difference in the 600–700-ms time window. In the 700–800- and 800–900-ms time windows, on the other hand, there was a modality difference in the incongruent condition, but not in the congruent condition. In other words, whereas incongruent olfactory stimuli only engender a positive slow wave in the 600–700-ms time window, incongruent visual stimuli engender a slow wave with a 600–900-ms latency. These findings suggest additional costs during the categorization of visual stimuli when distracted by the presence of incongruent odors, compared to vice versa. This asymmetrical interference is present in terms of a late and temporally extended interference period from 700 to 900 ms after target onset. These findings therefore complement the behavioral results in that they indicate substantial olfactory interference with visual categorizations.

#### Correlations Between ERP Amplitude and RT

**Figure 10:**
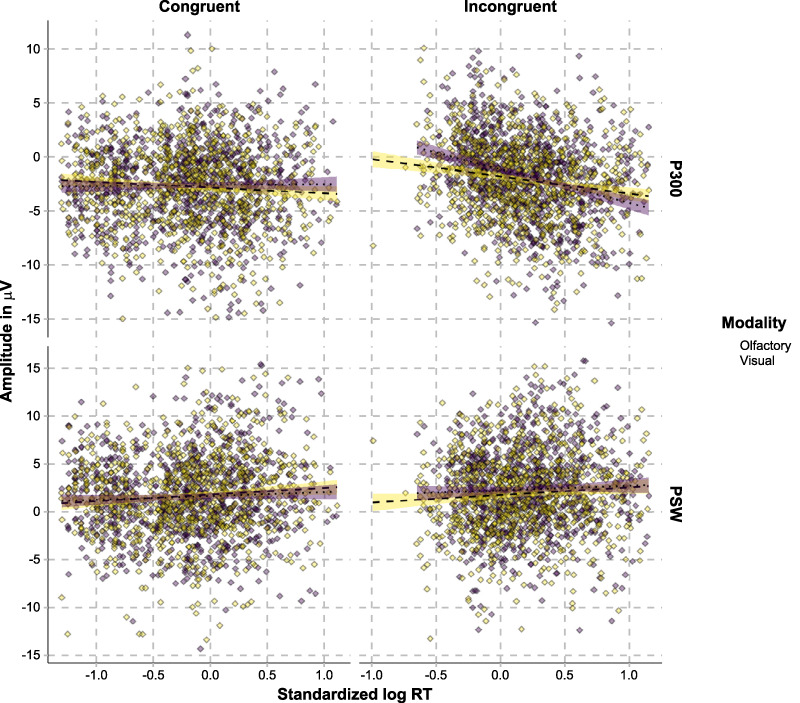
Correlations between mean ERP amplitudes in the P300 (CF ROI) and the PSW (CO ROI) time windows, differentiated on the basis of congruence and modality.

The relationships between single-trial mean ERP amplitudes and RTs in the P300 and the full PSW time windows are illustrated in Figure 10.

Our analysis in the P300 time window found that P300 amplitudes are unrelated to RTs in the congruent conditions (olfactory: β = −0.24, standard error [SE] = 0.24, CI = [−0.7, 0.23], *P* = 0.321; visual: β = 0.40, SE = 0.24, CI = [−0.07, 0.85], *P* = 0.094) but negatively correlated with RTs in the incongruent conditions (olfactory: β = −0.97, SE = 0.30, CI = [−1.55, −0.40], *P* = 0.001; visual: β = −2.21, SE = 0.33, CI = [−2.87, 1.57], *P* < 0.0001). Thus, the P300 effect engendered by incongruent stimuli is stronger in trials with fast responses. Under the assumption that P300 amplitude correlates with attentional demands needed to inhibit the interfering stimulus, this is to be expected. In trials were subjects are successful at inhibiting the interfering stimulus, RTs should be faster, and P300 amplitude should be higher due to the allocation of additional attentional resources for inhibition, resulting in a negative P300 amplitude–RT correlation. In order to further investigate whether this correlation also differs between modalities, we again performed a follow-up simple effects analysis. This was done by using a model with a log RT main effect predictor, the dummy-coded olfactory-congruent, visual-congruent, and olfactory-incongruent condition predictors, and their corresponding log RT × condition interaction terms. Here, the visual-incongruent condition serves as the reference condition against which the other conditions are compared. Any RT × condition effect entails that the ERP amplitude–RT association of those conditions differs from that of the visual-congruent condition. Strikingly, this analysis showed that the P300 amplitude–RT correlation is modality dependent, in that it is more pronounced in visual, compared to olfactory, categorization, β = −1.36, SE = 0.39, CI = (−2.14, −0.60), *P* = 0.0001. In other words, RTs during categorization of incongruent targets are to a greater extent predicted by P300 amplitude for visual than for olfactory stimuli. This in turn suggests that successful categorization of visual stimuli to a greater extent is dependent on attentional resources needed to inhibit the interfering olfactory percept. These findings further support the notion that incongruent odors interfere more with visual categorization than vice versa. We also conducted separate analyses of ERP amplitudes in the three PSW time windows (600–700, 700–800, and 800–900 ms). These found that PSW amplitude is unrelated to RTs in all of the conditions (see analyses in Supplementary Materials).

## Discussion

A well-established notion in psychological research is that visual stimulus processing “dominates” processing in other sensory modalities. Visual dominance has been suggested to involve asymmetric inhibition between sensory systems (e.g., Spence et al. 2012). If visual processes generally inhibit nonvisual processes, this should hold true also in categorization tasks with bimodal stimuli. We designed an odor–picture categorization task with cross-modal interference, testing the assumption of visual dominance using olfaction as a novel modality of comparison. Unexpectedly, our results provide converging behavioral and neurophysiological evidence for olfactory, rather than visual, dominance; incongruent odors exert more influence on visual processing than vice versa.

Our behavioral findings may provide new insights regarding the complimentary roles of olfaction and vision. In particular, when visual and olfactory inputs are congruent, visual processing indeed offers superior speed and accuracy, as would be expected by a purportedly dominant sensory system. But during incongruent input, it is instead olfactory cues that are surprisingly distracting, effectively impairing visual categorizations by disproportionally delaying responses and reducing their accuracy. The results of our study provide support for the notion that a foundational characteristic of olfaction is to effectively attract attention and processing resources in other senses. Early work (Herrick 1933) postulated that mammalian brain evolution was characterized by a rapid growth in visual, but not olfactory cortex, such that a key role for human olfaction would be to provide alerting cues for further visual processing. More recently, Köster et al. (2014) suggested that the olfactory system is particularly sensitive to contextually inappropriate odors, which might serve as an especially alerting cue to other senses, but is rather indifferent to expected odors. In a similar vein, our findings show a behavioral advantage for olfactory decisions only when odor and picture cues are incongruent. In other words, it is only when odors are inappropriate relative to the visual information that odors receive privileged processing. A possible objection to these conclusions may be that our experimental paradigm, employing a delayed response task, would not generalize to real-life situations where people can respond to stimuli directly. However, multisensory cues in ecological environments are arguably rarely perceived in exact synchronicity. Most real-life perceptual decisions instead require integration across sensory channels, as well as with motivational states, and are thus not instantaneous. Future variations of our experimental paradigm may explore the scope and limits of our observed results.

ERP effects provided valuable information about the cortical processing sequence underlying our behavioral observations. We were particularly interested in the idea that dominance stems from asymmetric inhibition between sensory systems (e.g., Spence et al. 2012). We reasoned that if asymmetric inhibition is responsible for sensory dominance, participants would require additional processing resources to compensate for the strong interference effect caused by incongruent input in the dominant modality. In line with this notion, we found a cortical processing sequence that started with overall effects of stimulus incongruency, gradually giving way to modality interactions that support our behavioral results of olfactory dominance. First, a more pronounced fronto-central P300 wave was observed during the categorization of incongruent stimuli. This effect was found to be negatively correlated with RTs and therefore to be stronger in trials with fast responses, in particular during categorization of incongruent visual stimuli. As the P300 amplitude is related to the mobilization of attentional resources during categorization tasks (e.g., Kok 2001), we interpret this P300 effect to reflect the additional attentional resources needed to inhibit the interfering percept in the incongruent condition. This interpretation is further supported by our observed negative RT–P300amplitude correlation in incongruent trials. In order to make a quick categorization decision, participants need to allocate additional attentional resources to inhibit the interfering percept. Fast responses during categorization of incongruent stimuli are therefore associated with higher P300 amplitudes, resulting in the observed negative correlation. Crucially, our findings show that this correlation is stronger for visual than for olfactory decisions, providing further support for asymmetric interference between visual and olfactory modalities; fast responses during categorization of incongruent visual stimuli are to a greater extent dependent on attentional resources needed to inhibit the interfering olfactory percept.

Our behavioral interaction effects were further reflected in a late, centro-occipital positive slow wave in the incongruent condition. Importantly, this effect had a later amplitude peak and longer latency for visual categorizations (peak 738 ms, latency 600–900 ms) than for olfactory categorizations (peak 673 ms, latency 600–700 ms). In other words, these findings provide evidence for olfactory dominance over visual input also in terms of differences in cortical activity. Positive slow wave activity has been found in tasks where target identification prompts the execution of additional tasks (García-Larrea and Cézanne-Bert 1998). In the congruent condition, the categorization response can be prepared already before the auditory target is presented. In the incongruent condition, on the other hand, the task consists of categorization based on the auditory target, selecting between visual and olfactory working memory representations, and finally making a categorization response based on the selected working memory representation. Positive slow wave activity has been linked to such response decision costs (Kok and De Jong 1980; Ruchkin et al. 1980; Ruchkin and Sutton 1983), response selection (Falkenstein et al. 1993, 1994), working memory updating operations prompted by a secondary task (Johnson and Donchin 1985), sustained attention to task performance (Gevins et al. 1996), or working memory load during retrieval (Honda et al. 1996; García-Larrea and Cézanne-Bert 1998). Thus, although several accounts of late positive slow waves have been suggested, including the proposal that they reflect the completion of a broad array of task-dependent cognitive operations following target detection (García-Larrea and Cézanne-Bert 1998), it seems clear that slow wave latency and amplitude reflect processing speed and processing effort. Our ERP findings therefore complement the behavioral results in that they show additional categorization costs for visual stimuli compared olfactory stimuli during cross-modal interference, thus indicating that incongruent olfactory stimuli interfere with visual categorization decisions more than vice versa.

It should be pointed out that our study differs from earlier studies of sensory dominance in that it investigates dominance in terms of inhibition between representations on the category level. Whereas earlier studies were concerned with mere stimulus detection (Sinnett et al. 2007; Koppen and Spence 2007a, 2007b, 2007c; Koppen et al. 2008), in some cases by using semantically meaningful stimuli (Koppen et al. 2008), the present study involved categorization of representations in incongruent contexts. In order to perform the task, participants were therefore required to activate two conceptual representations of the stimuli and to briefly hold those conflicting representations in working memory until the presentation of the auditory target. Rather than reflecting low-level asymmetric inhibition between sensory systems, our findings might therefore stem from asymmetric interference between working memory representations at the conceptual level. The observed categorization advantage of representations originating from olfactory input could result from a bias in the activation of the olfactory representations due to, for example, the focus on olfaction in the experimental context. A way of addressing this question would be to perform the same experimental paradigm, comparing visual input to input in another nonvisual (e.g., auditory) sensory modality. If such a study would find a categorization advantage for visual input, we would have more robust evidence for concluding that our findings of dominance of olfactory representations stems from differences between the olfactory and other nonvisual sensory systems. Further, our study does not test whether asymmetric inhibition also holds for within-category incongruencies (e.g., the smell of lilac interfering with the categorization of a picture of rose). Investigating this further could potentially shed more light on the question of whether our findings stem from low-level sensory inhibition or interference between higher-level, conceptual representations. Findings showing that the olfactory dominance effect is restricted to between-category incongruencies would suggest that asymmetric inhibition operates on the conceptual level, involving interference between categorical representations. Conversely, findings showing that the effect also applies to within-category incongruencies would indicate that inhibition occurs on lower levels of representation, involving interference between perceptual objects or sensory-level representations. Future research could also test whether olfactory input would have a similar influence on nonvisual (e.g., auditory) processing, thereby addressing the question of whether olfactory dominance stems from asymmetric inhibition between the olfactory and nonolfactory sensory systems more generally.

In this study, we have made the assumption that any observed differences in behavioral results between congruent and incongruent conditions reflect asymmetric “interference” among incongruent percepts. However, the observed congruence effects on the behavioral results could also, theoretically, stem from asymmetric “facilitation” in the congruent conditions (similarly to, e.g., Gottfried and Dolan 2003; Olofsson et al. 2013). That is, visual categorization could be facilitated by congruent olfactory information, more than vice versa. Importantly, both interference and facilitation would support the notion that olfaction has disproportional influence on visual processing, which was our a priori operationalization of olfactory dominance. Future work, however, could include visual and olfactory unimodal control conditions, against which it is possible to evaluate both interference and facilitation effects in the incongruent and congruent conditions, respectively. We speculate that, given our experimental design, we would be unlikely to observe any differences between unimodal and multimodal congruent conditions. In the congruent condition, participants can make their response selection directly after the stimuli is presented, and they have ample of time (1000–2000 ms) to categorize the stimuli before it is presented. It is therefore unlikely that their response decision would critically benefit from the information being multimodal (see Supplementary Materials for further discussion).

Another potential concern in this study is whether our observed modality differences could be explained in terms of a modality difference in stimulus similarity. A high degree of between-category stimulus similarity should make it harder to accurately differentiate between the cue stimulus and the stimuli of the other category (e.g., differentiating lemon odor from lavender/lilac), rendering the categorization task more difficult. In order to control for a potential confound, we included a participant-specific similarity index as a covariate in all of our statistical models that was based upon between-category stimulus similarity ratings (see Supplementary Materials). This covariate had zero effect in any of our models; thus, our results does not seem to have been affected by a modality difference in perceived stimulus similarity.

Further, in the present study, we did not measure respiration and could not explore possible differences in sniffing activity between congruent and incongruent conditions. Crucially though, since the auditory target informing about the categorization modality was presented 1–2 s after stimulus presentation, we have no reason to assume that there should be a difference in respiration patterns between these conditions. Prior work on similar paradigms also showed no such respiration differences (Olofsson et al. 2014).

Our findings clearly show that, contrary to the widely held notions in neurocognitive perception research, olfactory representations can dominate visual representations under conditions of equal task relevance. While the notion of visual dominance is well-established (Colavita 1974; Sinnett et al. 2007; Koppen and Spence 2007a; Koppen et al. 2008; Hartcher-O’Brien et al. 2010; Spence et al. 2012) and olfaction was traditionally viewed as a “primitive” or underdeveloped sense (see Laska 2015; McGann 2017 for reviews), our findings, along with recent cross-cultural evidence (Majid et al. 2018), provide reasons to reconsider such generalizations.

## Conclusions

This study shows that, contrary to the widely held notion of visual dominance, olfactory processing can dominate visual processing under conditions of equal task relevance and when differences in sensory encoding speed are controlled for. It extends earlier research by investigating cross-modal interference on the category level, using a categorization task with cross-modal interference. As such, it provides a novel paradigm for perceptual interference and competition for processing resources across sensory systems. Our findings provide support for the notion that visual behavioral categorization is especially disrupted by contextually incongruent odors. We speculate that incongruent odors might be particularly alerting, thereby attracting further processing in other senses.

## Funding

Swedish Foundation for Humanities and Social Sciences (M14-0375:1 to M.L.); Knut and Alice Wallenberg Foundation (KAW 2016:0229 to J.K.O.). *Conflict of Interest*: None declared.

## Supplementary Material

Supplementary_materials_bhaa050

## Footnotes

1 See Supplementary Materials for an extended discussion regarding the neurophysiological processes underlying the experimental task and the predicted ERP effects.

2 No precise chemical information regarding these products are provided by the manufacturers.

3 Before averaging, we included a dummy channel with zero activity that was removed after averaging. This is done in order to avoid rank deficiency, which can affect the subsequent ICA decomposition.

4 That is, by coding the variable as 0 or 1 and subtracting the mean, see, for example, Gelman and Hill (2006), p. 55.
