# Supplementary material for: Olfactory Influences on Visual Categorization: Behavioral and ERP Evidence
Source: Cereb Cortex. 2020 Mar 30;30(7):4220–37. doi: 10.1093/cercor/bhaa050 (PMC7264693; doi:10.1093/cercor/bhaa050)
Supplement: Supplementary_materials_bhaa050 [file supplementary_materials_bhaa050.pdf]

Supplementary materials

**Theoretical background and experiment design**	**2**
**Predicted ERP effects time-locked to visual-olfactory cue**	**2**
**Predicted ERP effects time-locked to auditory target**	**4**
**Power analyses**	**5**
**Prior research on the processing speed in visual and olfactory perception**	**7**
**Odor presentation time measurements**	**7**
**Identification and rating tasks**	**9**
**Statistical models**	**10**
**Analyses of ERP data**	**13**
**Model Results**	**14**
**Stimulus presentation ERP effects**	**25**
**References**	**27**

Theoretical background and experiment design

In order to shed light on the neurophysiological processes underlying visual-olfactory stimulus categorization under cross-modal interference, we recorded EEG measures and investigated ERP responses that were time-locked to the bimodal cue presentation, and to the presentation of the auditory target. Since this is the first study of its kind to include ERP data, we were cautious in our predictions in terms of specifying exact effects, time windows and regions of interest. We can only hypothesize which ERP outcomes to expect on the basis of somewhat related studies investigating, for example, working memory and categorization of stimuli in other modalities. In the following, we briefly outline the assumed cognitive requirements of the experimental task, and then provide an overview of the ERP effects that could be expected on the basis of these assumptions.

Since subjects maintain and categorize bimodal stimuli over a brief retention period, the experimental task can be regarded as a working memory task. When the olfactory and visual stimuli are congruent, the task is simple. Participants need to map the olfactory and visual sensory input to a multimodal semantic representation of the object at hand (e.g., lilac), and thereby determine that the stimuli match. They can then categorize the object as fruit or flower, maintain this information in short-term memory, and wait for the auditory target. The auditory target therefore only functions as cue for their response, but does not provide any additional information regarding which response is correct.

When the stimuli are incongruent, on the other hand, the task is more difficult. Participants need to establish two (olfactory and visual) object representations to be encoded and retained in working memory simultaneously, but that compete for attentional resources. In order to make a categorization decision, participants then need to integrate the information provided by the auditory target with the information retained in working memory. The object representation corresponding to the target needs to be categorized as fruit or flower. A response decision can then be made on the basis of this information (see Figure A1). However, during this process, the conflicting object representation of the competing sensory modality needs to be resolvd, either passively (by a dominant stimulus overshadowing the non-dominant stimulus) or actively (by a dominant stimulus inhibiting the non-dominant stimulus). If overshadowing/inhibition is asymmetric across the visual and olfactory channels, more attentional resources are therefore required during the categorization of the non-dominant, (presumably olfactory) object, then during the categorization of the dominant (presumably visual) object, in order to compensate for the interference advantage of the visual object over the olfactory object. As illustrated in Figure A1, we assume that interference between sensory systems may occur both during encoding, directly following stimulus presentation, and at retrieval, following the presentation of the auditory target. However, it is only at the latter time-point - when information regarding the target modality is availible- that any cortical differences related to asymmetric inhibition between sensory modalities will be observed.

Predicted ERP effects time-locked to visual-olfactory cue

At the time point of stimulus presentation, participants are presented with either a congruent or an incongruent odor-picture pair. Many ERP studies have investigated cortical responses to both linguistic and non-linguistic incongruent information. It has been found that semantically incongruent words engender a centro-parietal negative shift, peaking around 400 ms after word presentation, called the N400 effect (see Kutas and Hillyard 1980 for initial evidence; and Kutas and Federmeier 2011 for a review). Other studies have also found a similar response to semantically incongruent pictures presented in a sentence context (Nigam, Hoffman and Simons 1992), incongruent pictures and sounds (Gallagher et al. 2014; Kovic, Plunkett and Westermann 2009, 2010), and incongruent visual scenes

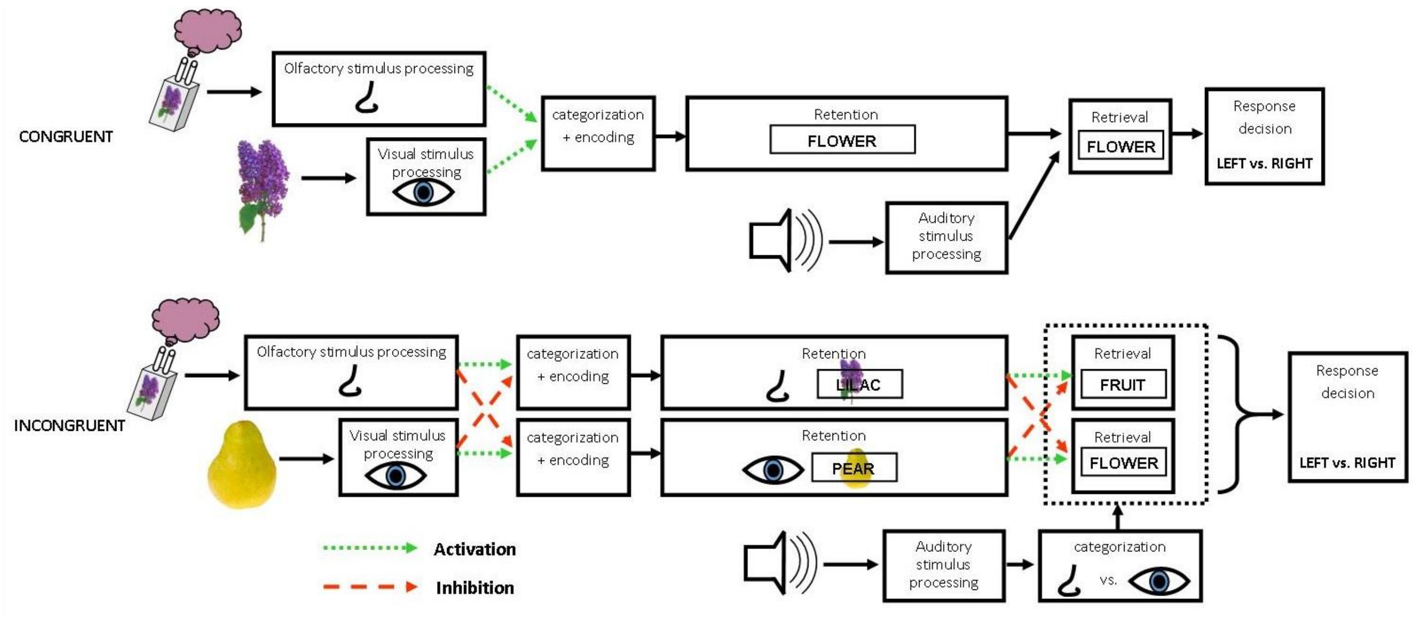

Figure A1. An illustration of the cognitive processes assumed to underlie visual dominance in the delayed response odor-picture categorization task with cross-modal interference. When stimuli are congruent (e.g., lilac odor and lilac picture), the task is easy. Participants process the olfactory and visual sensory input and map this information to a multimodal object representation that is categorized either as fruit or flower directly. This information is encoded into working memory until the presentation of the auditory target, at which time point it is used to make a response decision. However, when stimuli are incongruent (e.g., lilac odor and pear picture), the task is hard. The sensory input needs to be mapped to seperate olfactory and visual object representations that are encoded and retained in working memory simultaneously, and that compete for attentional resourses. These representations are then integrated with the information provided by the auditory target. The object representation that corresponds to the category of the auditory target (i.e., olfactory or visual) is retrieved from working memory and categorized as fruit or flower. During this process, the conflicting object representation of the competing sensory modality needs to be resolved, by overshadowing or inhibition. If baseline inhibition between sensory systems is asymmetric, more attentional resources are therefore required during the categorization of the non-dominant (presumably olfactory) object, in order to inhibit the dominant (presumably visual) object, then during the categorization of the dominant object, which requires less inhibition of the non-dominant object.

(Mudrik, Lamy and Deouell 2010). Importantly, a few studies have found an N400-like response to words or pictures that were semantically incongruent with a previously or simultaneously presented odor (Castle, Van Toller and Milligan 2000; Grigor, Van Toller, Behan and Richardson 1999; Kowalewski and Murphy 2012; Olofsson et al. 2014; Sarfarazi 1999; but see Robinson, Reinhard and Mattingley 2015 for conflicting results).

In many of these studies, the effect is different from the traditional, “linguistic” N400, in that it has long latency, often lasting throughout the full epoch (e.g., Grigor et al. 1999; Kowalewski and Murphy 2012; Mudrik et al. 2010; Sarfarazi 1999) and a more frontal scalp distribution (e.g., Gallagher et al. 2014; Grigor et al. 1999). A similar ERP incongruence effect, consisting of a centro-frontal negative deflection in the 350-550 ms time window, has been found in stroop tasks (Liotti, Woldorff, Perez and Mayberg 2000; Markela-Lerenc et al. 2004; Qiu, Luo, Wang, Zhang and Zhang 2006; Xiao, Dupuis-Roy, Yang, Qiu and Zhang 2014). A few studies have instead found a pareito-occipital positive incongruence effect in the 400-600 ms time window, called the P400 (Liu, Wang and Jin 2009; Puce, Epling, Thompson and Carrick 2007).

At the time point of stimulus presentation, we therefore expected - as stated in the preregistration - incongruent stimuli to engender either a negative or possibly a positive incongruence ERP effect with either a centro-parietal or centro-frontal scalp distribution, starting around 300-350 ms after stimulus presentation, and possibly extending throughout the epoch.

Predicted ERP effects time-locked to auditory target

ERP responses time-locked to auditory targets are of greater theoretical interest because, as mentioned above, it is only when participants have information regarding the target modality that differences related to asymmetric interference between sensory systems can be observed. Once this information becomes available, the object representation in the target modality can be retrieved and categorized at the expanse of the representation in the interfering modality. If interference is asymmetric, participants can be expected to mobilize additional attentional resources in order to be able to effectively inhibit the object representation of the dominant (presumably visual) modality.

Several studies have found a modulation of the frontocentral N2 wave, a negative deflection peaking between 200-350 ms post stimulus onset, in tasks that involve inhibitory control and/or monitoring of e.g. incorrect responses, such as in go/no-go tasks and the Eriksen flanker task (e.g., Bartholow et al. 2005; Berchicci, Spinelli and Di Russo 2016; Dong, Yang, Hu and Jiang 2009; Heil, Osman, Wiegelmann, Rolke and Hennighausen 2000; Nieuwenhuis, Yeung, van den Wildenberg and Ridderinkhof 2003; and Folstein and Van Petten 2007 for a review). In the present study, inhibitory control over the interfering object might be reflected by an increase in N2 amplitude in the incongruent conditions in comparison to the congruent conditions, in which no inhibition is required. Further, if between-modality interference is asymmetric, a greater deal of inhibitory control over the dominated modality might be required, resulting in a more pronounced N2 wave during the categorization of dominated olfactory stimuli.

Perhaps of greater significance is the previously mentioned P300 response, a positive, centro-parietal or in some cases a centro-frontal wave, that often peaks around 300 ms post stimulus presentation (e.g. Donchin 1981; Kutas, McCarthy and Donchin 1977; Picton 1992; Sutton, Braren, Zubin and John 1965; Verleger 1997; and Polich 2007, 2011 for reviews). Traditionally, the P300 is engendered by stimuli that deviate from an expected pattern (e.g. Kutas et al. 1977; Sutton et al. 1965), but it has also been observed in stimulus-driven, categorization tasks (e.g., Chen, Li, Qiu and Luo 2006) as well as in language comprehension tasks (e.g., Coulson, King and Kutas 1998; Hörberg, Koptjevskaja-Tamm and Kallioinen 2013; Keidel and Hörberg 2017) more generally. The P300 is usually differentiated into the P3a and the P3b subcomponents (e.g, Polich 2007, 2011). The P3a does not require active attention and has been linked to involuntary attention shifts (e.g., Kok 2001). The P3b, on the other hand, require active attention in that the eliciting stimuli must be task related (Kok 2001). In the following, we will therefore only focus on the P3b response, but will refer to it as "P300". The P300 has been suggested to reflect the updating of information in working memory (i.e., "context updating" see Donchin and Coles 1988) as a response to e.g. unexpected stimuli, but also to reflect event categorization (Kok 2001), or, relatedly, processes that links stimulus categorization with a response decision (Desmedt 1980; Verleger 1988, 1997; Verleger, Jaśkowski and Wascher 2005) such as inhibition of unrelated activity in order to focus attention on the task at hand (Polich 2007). According to Kok (2001), event categorization involves matching between external stimuli and internal, working memory representations, a process that is mediated by attention. Importantly, P300 amplitude is on this view assumed to reflect the amount of attentional resources that are invested into the categorization task (e.g. Kok 2001), which, in turn, co-occurs with inhibition of task-unrelated activity (Polich 2007). In the context of the present study, the additional attentional resources needed to inhibit the interfering sensory object during the categorization of incongruent stimuli can therefore be expected to result in an enhanced P300 wave, when compared to the categorization of congruent stimuli during which cross-modal interference is not an issue. Further, more attentional resources might be required to inhibit the sensory object in the dominant, visual modality, than the sensory object in the dominated, olfactory modality. This should result in a more pronounced P300 wave during olfactory object categorization in comparison to visual object categorization.

At the time point of the presentation of the auditory target, we therefore expect, as stated in our preregistration, incongruent trials to engender an enhanced N2 wave with a central scalp distrbution and an enhanced P300 wave with either centro-parietal or centro-frontal scalp distribution in

comparison to congruent trials. Further, if there is any evidence for a visual dominance effect in the behavioral results, we also expect both the N2 wave and P300 wave to be more pronounced during the categorization of incongruent olfactory objects then during the categorization of incongruent visual objects.

Power analyses

Prior to running the experiment, we conducted power analyses of RT differences and mean P300 amplitudes in auditory target ERPs. These analyses were done on the basis of pilot data and simulation. We piloted the experiment on four participants and used that data in order to get a rough estimate of the model parameters in generalized mixed effects models analysing RTs and P300 amplitudes (see below for model details). We then generated simulated data sets of different sizes (N=10, 20, 30, 40) using some of the model parameters, but varying the effect sizes of the Modality effect and the Congruence × Modality interaction effects (standardized $\beta = 0.1, 0.2, 0.3, 0.4$). For each sample size–by-effect-size combination, we simulated 1000 data sets, and fitted a mixed effects model on each of those data sets. Power of each beta coefficient was then calculated as the percentage of times that the coefficient at hand was significant. Power calculations were conducted in R. The R code with the power analysis are available at osf.io/7qnwu/. The results of the RT power analyses are shown in Figure A2 (power of each coefficient) and A3 (mean and 95% percentile of the Congruence and Congruence × Modality betas), and results of ERP power analyses are shown in Figure A4 (coefficient power) and A5 (mean and 95% percentile of the Congruence and Congruence × Modality betas).

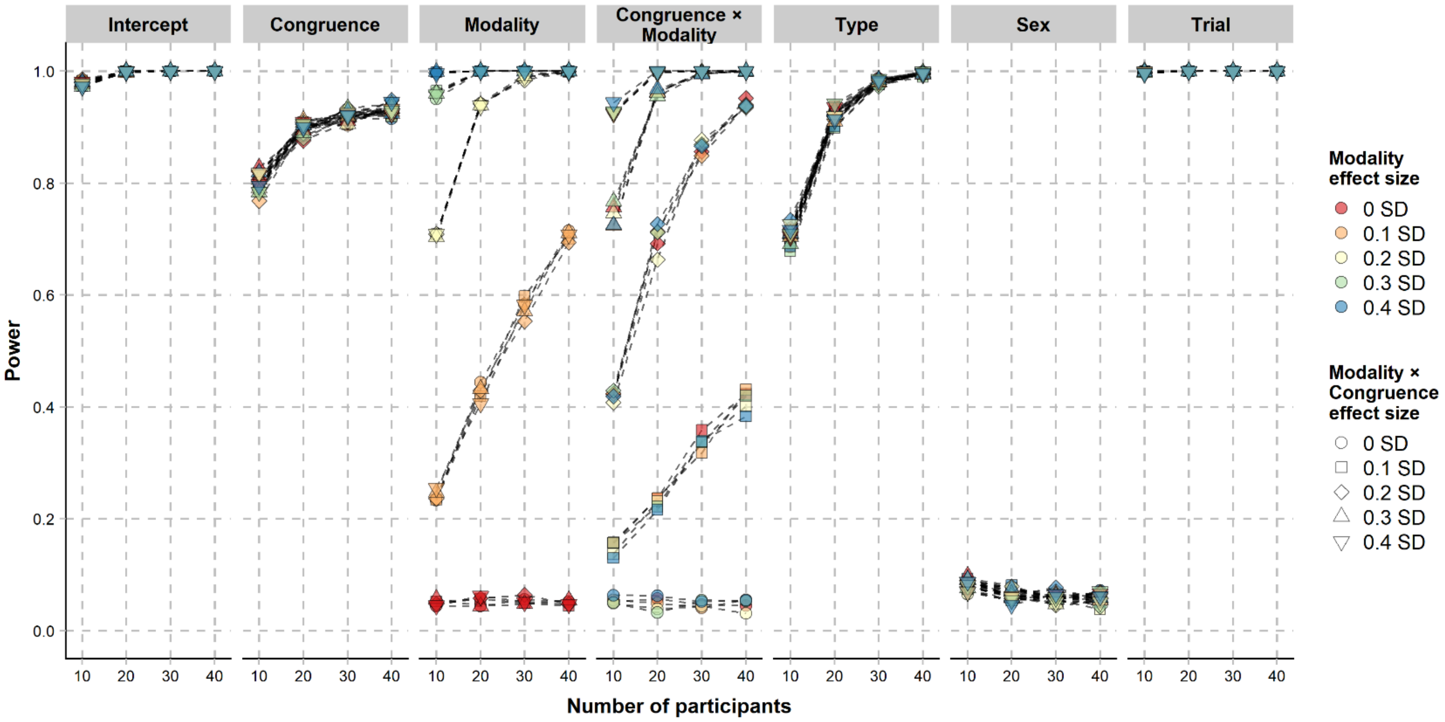

Figure A2. Results of simulation-based power analyses of RT data for each model coefficient, differentiated on the basis of number of participants (10, 20, 30 or 40), Modality effect size, and Modality × Congruence effect size.

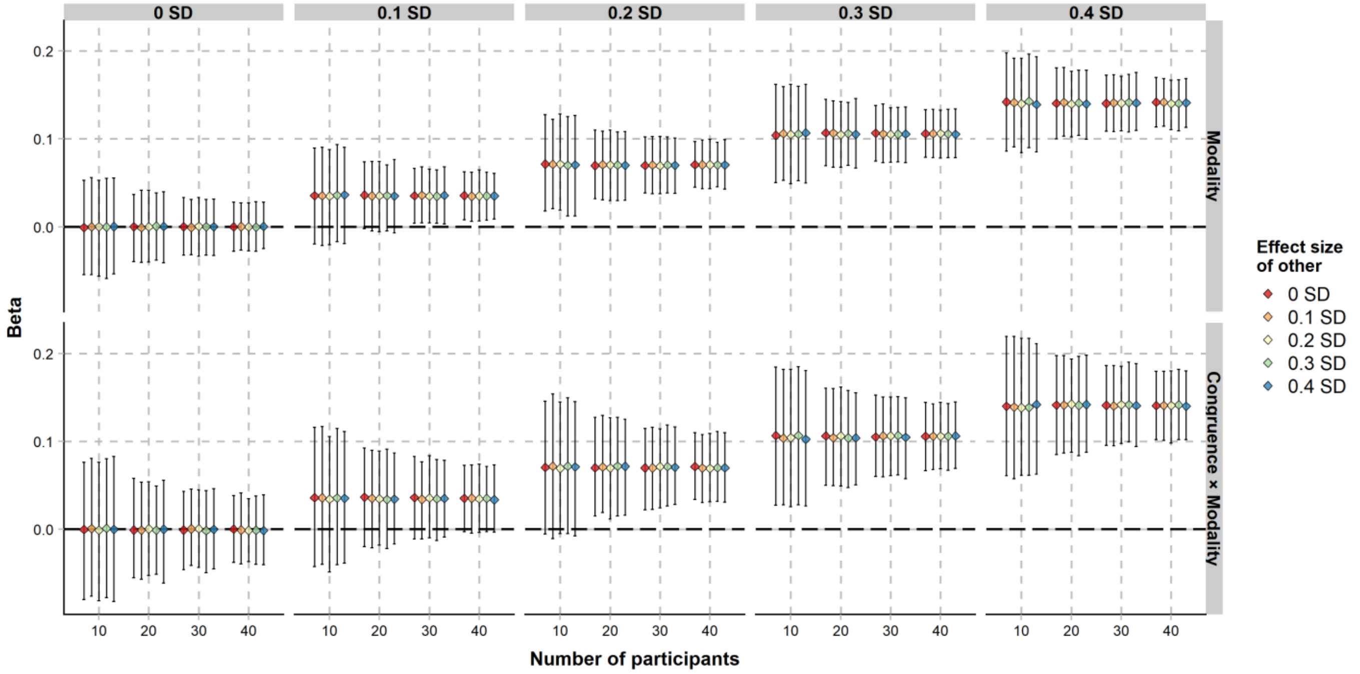

Figure A3. Results of simulation-based power analyses of RT data, showing mean and 95% percentile of the Congruence and Congruence × Modality betas, differentiated on the basis of number of participants (10, 20, 30 or 40), Modality effect size, and Modality × Congruence effect size.

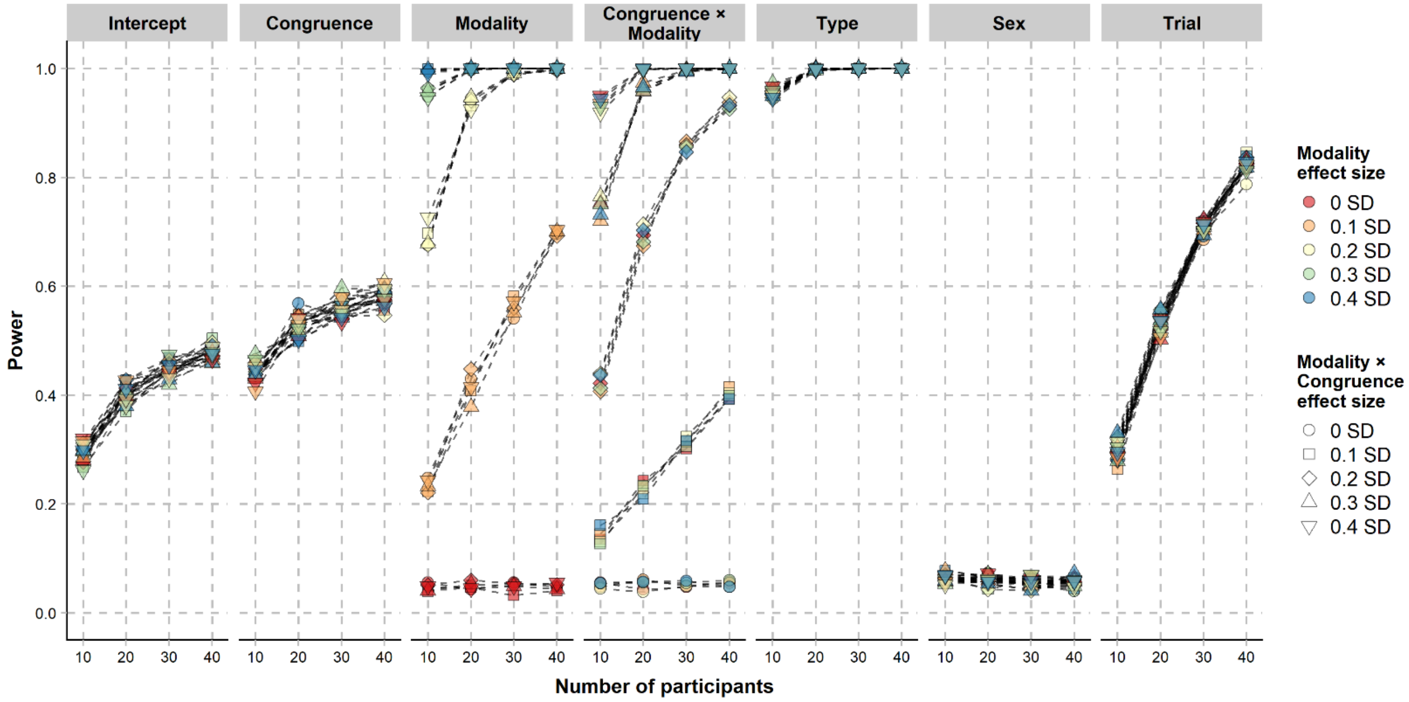

Figure A4. Results of simulation-based power analyses of ERP data for each model coefficient, differentiated on the basis of number of participants (10, 20, 30 or 40), Modality effect size, and Modality × Congruence effect size.

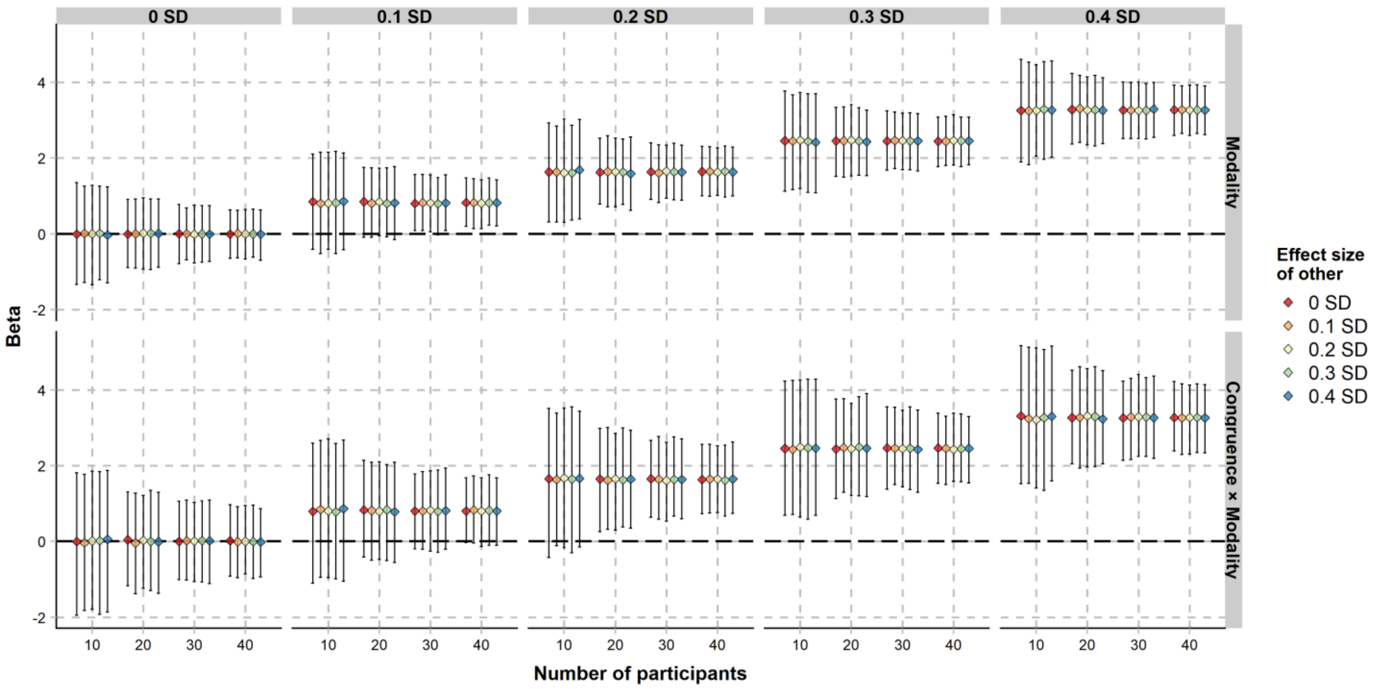

Figure A5. Results of simulation-based power analyses of ERP data, showing mean and 95% percentile of the Congruence and Congruence × Modality betas, differentiated on the basis of number of participants (10, 20, 30 or 40), Modality effect size, and Modality × Congruence effect size.

Prior research on the processing speed in visual and olfactory perception

A general behavioral response speed advantage of visual processing over olfactory processing can be expected, independent of whether crossmodal, visual-olfactory interference is symmetric or not. Whereas visual detection reaction times (RTs) are on average about 300 ms (e.g., Amano et al. 2006; Collins and Long 1996), a few studies have found olfactory detection RTs around 800 ms (Olofsson 2014; Olofsson, Bowman and Gottfried 2013), indicating a 400 ms processing time difference. Also ERP latencies engendered during visual and olfactory processing differ with about 300-400 ms, In an olfactory oddball task (i.e., measuring the neurophysiological response to odors deviating from an expected pattern), Pause et al. (1996) found the olfactory N1, P2 and P300 components to peak at roughly 450, 600 and 830 ms, respectively. This is about 350-400 ms later than the corresponding visual ERP components engendered in visual oddball tasks (e.g. Alexander et al., 1995; Geisler & Polich, 1994; Romero & Polich, 1996). Taken together, these findings are highly suggestive of a 350-400 ms delay in olfactory processing time.

Odor presentation time measurements

In order to estimate the timing of odor presentation with respect to the on-screen visual presentation of the sniff cue, we performed measurements of the temporal performance of the olfactometer in a similar experimental set up as in the original experiment. In this session, we measured the activity of the olfactometer with respect to the opening of the odor channel valve, the on-screen visual presentation of the odor cue, and the concentration of odor molecules in the olfactometer output airflow.

Olfactometer activity was measured via a trigger signal whose output changes when an odor channel is activated or deactivated. The presentation of the visual cue was measured with a phototransistor circuit that was attached to the upper left corner of the computer screen, where a black rectangle appeared simultaneously with the visual cue. The electric potential of the output of the circuit is proportional to the illuminance of a small area of the screen directly under the phototransistor. The concentration of the emissions from the olfactometer were measured with a photo-ionization detector (PID; miniPID 200B, Aurora Scientific Inc, Aurora, Ontario, Canada) with a rise time of 0.6 ms, a detection limit of

0.1 ppm (propylene in air) and a ionization energy of 10.6eV. The PID measures volatile organic compounds and other gases. As compounds enter the detector they are exposed to high-energy UV photons and are ionized when absorbing the UV light, resulting in the formation of positively charged ions which produce an measurable electric current. The current is proportional to the number of ions and therefore to the concentration of the substance. Measurements are restricted to molecular structures that have similar or lower ionization potentials than the photons produced by the PID. The phototransistor circuit, the olfactometer output trigger signal, and the PID were connected to the analog inputs of a Measurement Computing USB-1608FS-Plus data acquisition device[1], measuring all three signals at a sampling rate of 2kHz. Using a windows 10 laptop, the data was collected in Python (Python 3.7) using the mcculw package, which is the official application programming interface (API) for the data acquisition device.

We performed measurements with the continuous air flow set to 0.5 l/m and an individual channel air flow of 2.5 l/m, as in the experiment. We used limonene (CAS 5989-27-5) as odor chemical. Limonene has an ionization potential low enough to be measured by the PID (~8.2 eV) and is perceptually similar to the lemon odor used in the experiment. Each measurement trial had a similar structure as in the experiment. Each trial started with a 1 second delay before the odor was presented. Following this delay, the computer signal for opening the odor channel valve was sent simultaneously with the presentation of the visual cue (a black fixation cross centered on the screen). After a 400 ms delay, the visual cue was replaced with an image. After another 1500 ms delay the signal for closing the odor channel valve was sent simultaneously as the image disappeared. The trial then continued until 10 seconds had passed. Thus, on each trial, odors were presented for a total of 1900 ms with a total inter-stimulus-interval of 8100 ms. We performed 20 measurement trials. However, since there is no baseline preassure in the system prior to the presentation of the initial odor, the initial trial was excluded from our analyses (in line with Lundström et al. 2010).

Across trials, we calculated the mean PID response at each time point. For each trial, we also estimated the time of the opening of the olfactometer valve, the time of presentation of the visual cue, the time of the onset of the odor output, and the time at which odor output reached 50%. The time of the opening of the valve was estimated on the basis of the electrical activity of the olfactometer. It is the first within-trial time point at which the electrical response is above three standard deviations of the within-trial mean electrical response. The time point of the visual cue is the first within-trial time point at which the phototransistor response is below 75% of the within-trial phototransistor response range. The time of odor output onset is the first within-trial time point at which the PID response exceeds 5 standard deviations from the PID response in a 1000 ms within-trial baseline time window. The time at which odor output reached 50%, finally, is the within-trial time point at which the PID response reaches 50% of the within-trial PID response range.

Figure A6 illustrates the mean PID response across time points. The time scale is referenced to the time of the onset of the visual cue (time 0). The vertical lines illustrate the mean time point of the onset of the opening of the olfactometer valve, the onset of the odor output, and the time point at which 50% odor output is reached. Shaded areas illustrate ±3 standard deviations from the means. On average, the olfactometer valve opens 5.4 ms after the presentation of the visual cue. However, the standard deviation of the time of the opening of the valve is 6.7 ms. Thus, across trials, the valve opens within the same time range as the visual cue is presented. On average, the odor is presented about 53.5 ms after the visual cue has been presented (standard deviation: 6.7 ms), and reaches 50% output around 217.5 ms after the presentation of the visual cue (standard deviation: 17.9 ms).

[1] The certificate of calibration for the data acquisition device is 5685368

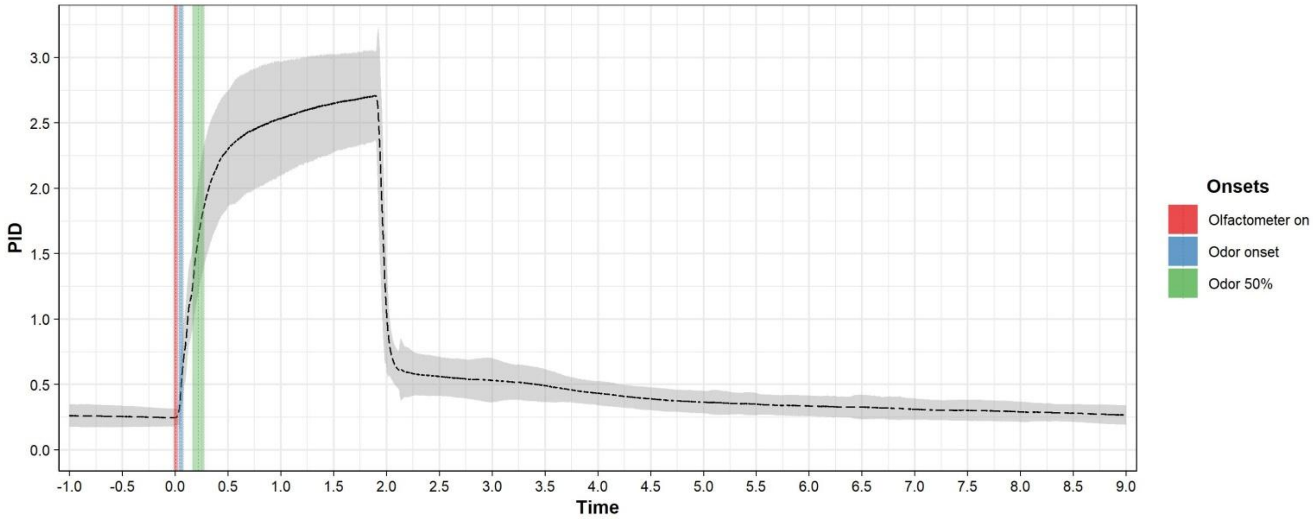

Figure A6. Mean PID response across time points, referenced to the time point of the onset of the visual cue. Vertical lines illustrate the onset of the opening of the olfactometer valve, the odor output onset and 50% odor output. Shaded areas show ±3 standard deviations from the mean.

Identification and rating tasks

Before the experiment started, participants performed an identification task and two stimulus rating tasks. The odor presentation procedure was the same in all three tasks. First, a black fixation cross appeared for 1500 ms in the center of the screen, indicating that an odor was about to be presented and that it was time to prepare for the sniff by inhaling. Following this inhalation cue, a blank screen was presented for 200 ms. Then, a red fixation cross appeared that indicated that it was time to sniff, simultaneously as the odor was presented in the olfactometer.

In the identification task, participants were exposed to the four odors in the manner described above. They identified each odor by selecting one of the four images that were presented simultaneously in a 2 × 2 grid on the screen. Odor presentation order was counterbalanced across participants. Image positions were randomized within each trial. Each trial was repeated until it was answered correctly. Each odor was identified twice in order for participants to get familiarized with the stimuli.

In the first rating task, participants rated each odor quality in terms of intensity, pleasantness, specificity (i.e., the extent to which the odor provided a good match with the object indicated by its label), and edibility, using a scale from 1 to 100. Following odor presentation, the four rating scales appeared on the screen. Participants rated odors by moving a marker across a graphical illustration of each scale using the mouse and then clicking the numerical rating value that was shown directly below each scale. Both odor and scale order was counterbalanced across participants.

The results of the odor quality rating task is illustrated in Figure A7 below. As shown in the figure, all odors are rated fairly similarly in terms of intensity, specificity and pleasentness. As can be expected, pear and lemon are rated as high in edibility, and lavender and lilac as low.

In the second rating task, each stimulus was rated for its between-category similarity, using a scale from 1 to 100. That is, all stimuli were rated with respect to how perceptually similar they are to the two stimuli of the other category (e.g., the similarity between the pear odor, on the one hand, and the lavender and lilac odors, on the other). Each rating trial begun with a 2000 ms presentation of a text, centered on the screen, indicating the modality and names of the stimuli to be rated (e.g., “Pictures pear and lavender”). On olfactory trials, the two odors were then presented sequentially in the manner described above, with a 2000 ms blank screen in between the two presentations. On visual trials, a black fixation cross was presented for 1000 ms, followed by a 1500 ms presentation of the first image,

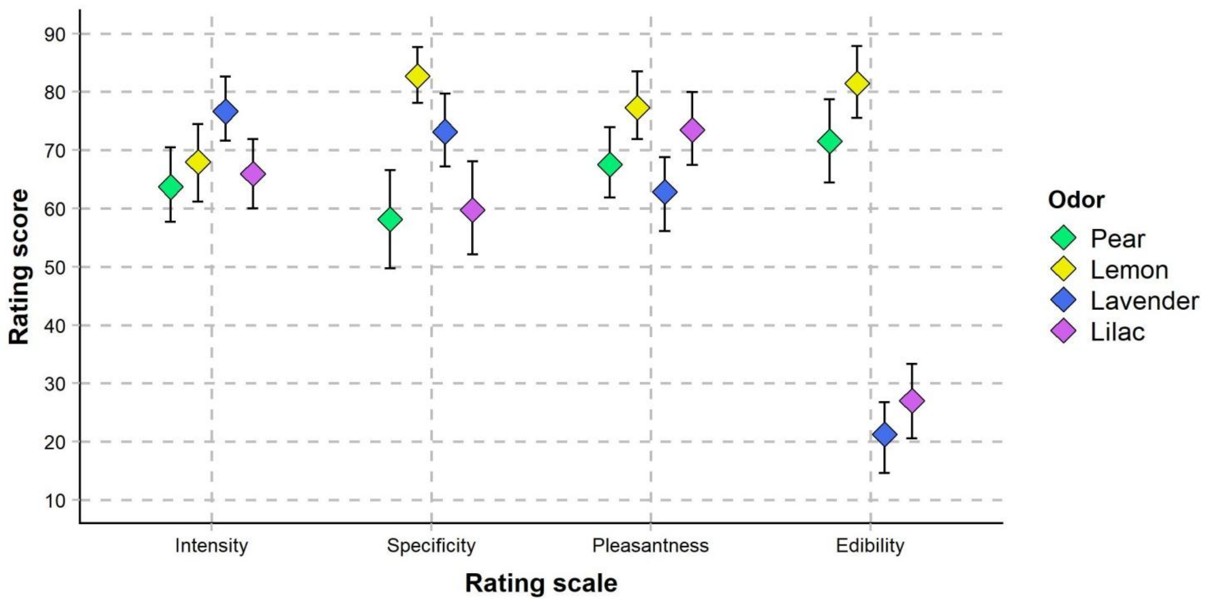

Figure A7. Mean odor ratings in terms of intensity, specificity, pleasantness and edibility. Error bars show 95% confidence intervals, calculated on the basis of bootstrapping.

another 1000 ms presentation of the fixation cross, and, finally, a 1500 ms presentation of the second image (everything being presented in the center of the screen). Following stimuli presentation, a rating scale identical to that of the first rating task was presented in the center of the screen. Each object pair was rated twice. Each participant therefore performed a total of 2 (modality) × 4 (stimuli) × 2 (cross-category stimuli) × 2 (repetitions) = 32 rating trials. Object pair presentation order was pseudo-randomized, with the following exceptions. The first occurrence of an object pair always appeared among the 16 initial trials and the second occurrence among the 16 final trials. The within-trial presentation order of an object pair was reversed on its second repetition. The presentation order of this within-trial ordering (e.g., whether pear-lavender appeared before or after lavender-pear) was counterbalanced across participants and modalities. All rating trials were always preceded and followed by trials with stimuli in the opposite modality (e.g., olfactory trials always being preceded and followed by visual trials). The modality of the initial trial (i.e., the first trial being visual or olfactory) was counterbalanced across participants. Mean similarity ratings, corrected for between-participant variation by standardizing ratings within participants, are shown in Figure A8. As the figure illustrates, odors are consistently rated as more similar to each other than pictures, thereby introducing a possible confound into the experiment. In order to control for this possible confound, we calculated a stimulus similarity index that was included as a covariate in all statistical analyses. First, ratings were standardized within participants, ensuring that participant rating means and standard deviations were the same for each participant. The between-category similarity index was then calculated within each participant, cue stimulus and modality as the mean of the standardized similarity ratings involving the stimulus at hand. Since participants rated the stimuli on their similarity with each of the two within-category stimuli twice (e.g., two similarity ratings of lemon-lilac, and two of lemon-lavender), this index was the mean of four ratings. The mean similarity index is illustrated in Figure A9.

Statistical models

All data was analysed with Bayesian mixed effects modeling in the Stan modeling language (Stan Development Team 2017), using the R package Rstan (Stan Development Team 2018), as well as with frequentist mixed effects modeling. Frequentist linear mixed effects models were fitted with the lmerTest() package (Kuznetsova, Brockhoff and Bojesen Christensen 2014), in which degrees of freedom for the calculation of p-values are estimated with Welch-Satterthwaite approximation. Frequentist logistic mixed effects models were fitted with the glmer() function in the lme4 package

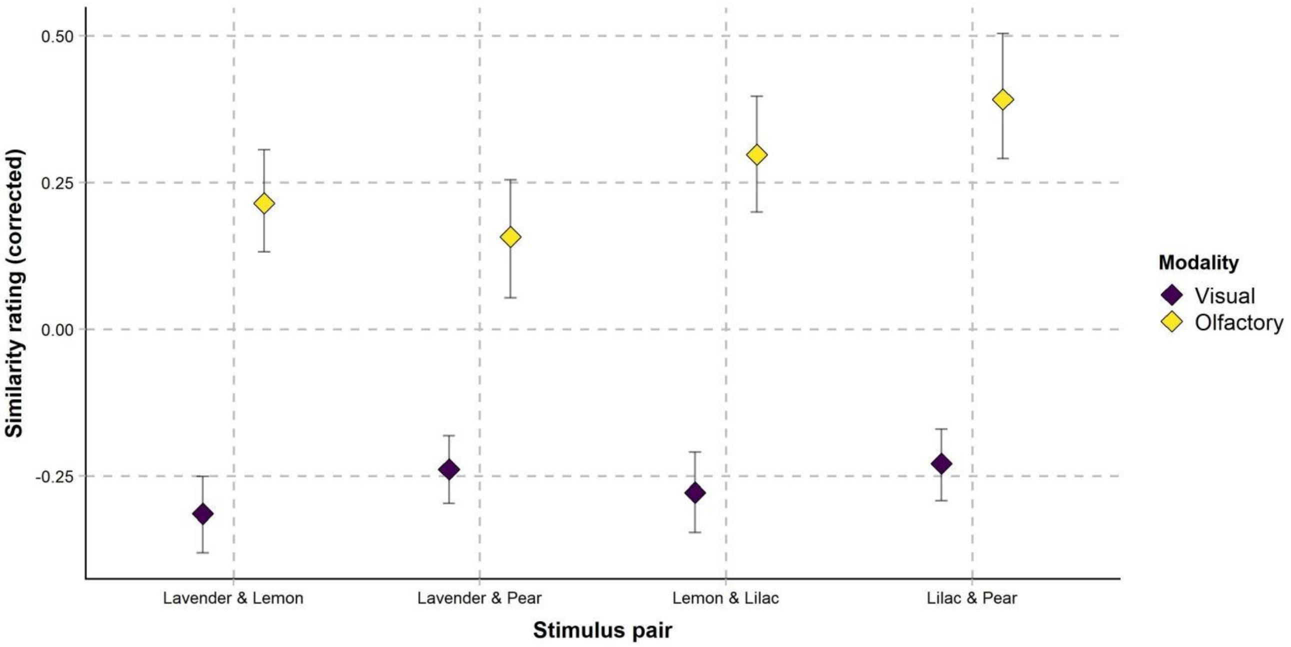

Figure A8. Mean similarity ratings, corrected for between-participant variation, for each stimulus pair in each modality. Error bars show 95% confidence intervals, calculated on the basis of bootstrapping.

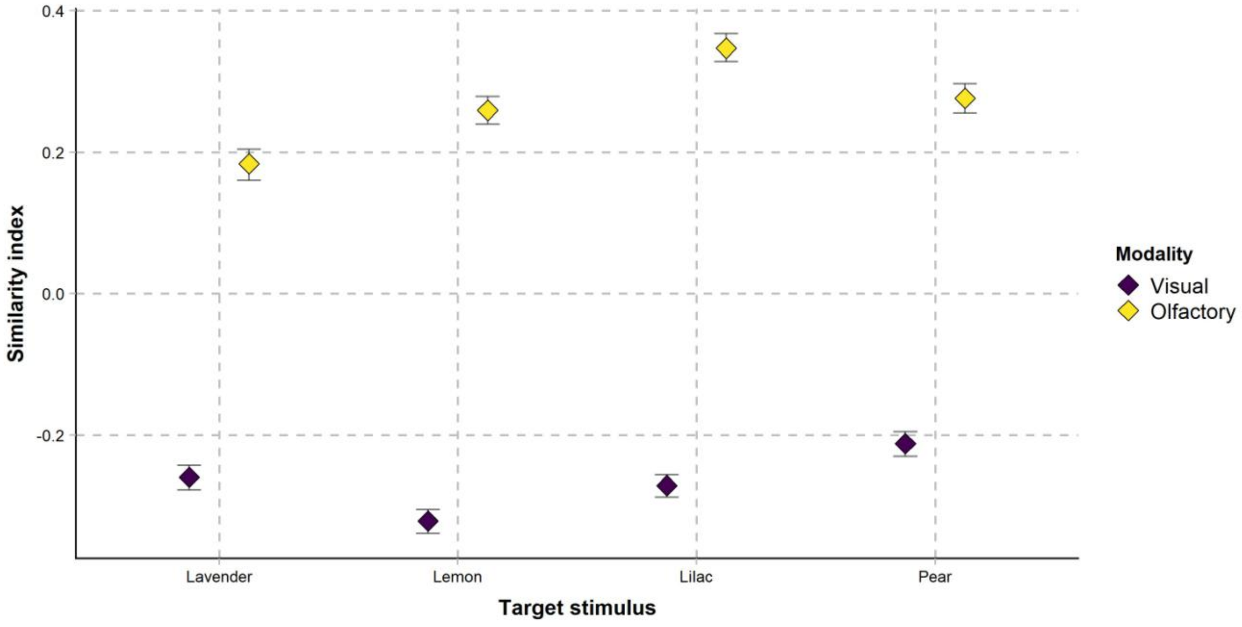

Figure A9. Mean similarity index for each stimulus type in each modality. Error bars show 95%, calculated on the basis of bootstrapping.

(Bates, Maechler, Bolker and Walker 2014). RTs and ERP amplitudes were analyzed with linear mixed effects modeling, and accuracy with logistic mixed effects modeling.

In the pre-registration, we specified that inferences would be made on the basis of Bayesian model comparison using Bayes factor. Bayes factor is the ratio of the likelihood probability of two competing models or hypotheses. It expresses the likelihood of the data given a statistical model relative to the likelihood of the data given a null model (e.g. Wagenmakers et al. 2018). A Bayes factor > 3 is usually considered as evidence for a model (Jeffreys 1961; Wagenmakers et al. 2018). In our case, inferences about the effect of a parameter (e.g., the Congruence × Modality interaction) can be done by

comparing a model with the parameter at hand against an identical model without that parameter. However, Bayes factor is highly sensitive to the choice of prior (Gelman and Robert 2013; McElreath 2016). Weakly informative or improper parameter priors can lead to ill-defined Bayes factors that always provide evidence for the null model, independent of the data (Bartlett 1957; Gelman and Rubin 1995; Strachan and van Dijk 2005). In our case, Bayes factor analyses turned out to be too conservative for our complex models in the sense that they were inconsistent with the parameter statistics as well as with the results of the frequentist model comparisons. Therefore, we instead base our conclusions on the basis of the parameter statistics, considering a parameter 95% credibility interval (CI) not including zero as evidence for an effect of the parameter at hand. We also report the posterior probability of a parameter being zero or taking on values in the opposite direction of the mean parameter estimate. Nevertheless, for transparency, below we report all results of the statistical analyses, including Bayes factor analyses and frequentist model comparisons.

The models of stimulus presentation ERPs included fixed effects of Congruence (Congruent vs. Incongruent), Object Category (Fruit vs. Flower), the Congruence × Object Category interaction, Gender, Trial number and Similarity index. Thus, mean stimulus presentation ERP responses y for the i:th participant and the j:th item were modelled as

$$y_{ij} = \alpha_0 + \alpha_i + \alpha_j + \beta_i \mathrm{Trial}_{ij} + \beta_1 \mathrm{Congruence}_{ij} + \beta_2 \mathrm{Category}_{ij} + \beta_3 \mathrm{Congruence}_{ij} \times \mathrm{Category}_{ij} + \beta_4 \mathrm{Gender}_{ij} + \beta_5 \mathrm{Trial}_{ij} + \beta_6 \mathrm{Similarity}_{ij} + \varepsilon_{ij},$$

$$\varepsilon_{ijk} \sim N(0, \sigma^2(\varepsilon_{ij})),$$

$$\begin{pmatrix} \alpha_i \\ \beta_i \end{pmatrix} \sim N\left(\begin{bmatrix} 0 \\ 0 \end{bmatrix}, \begin{bmatrix} \sigma^2(\alpha_i) & \sigma(\alpha_i, \beta_i) \\ \sigma(\beta_i, \alpha_i) & \sigma^2(\beta_i) \end{bmatrix}\right),$$

$$\alpha_{ij} \sim N(0, \sigma^2(\alpha_{ij}))$$

All other models contained fixed effects of Congruence, Modality (Visual vs. Olfactory), the Congruence × Modality interaction, Object Category, Gender, Trial number, Delay, Similarity index. Thus, log RTs, accuracy as expressed in log-odds, and mean auditory target ERP responses for the i:th participant and the j:th item were modelled as y accordingly

$$y_{ij} = \alpha_0 + \alpha_i + \alpha_j + \beta_i \mathrm{Trial}_{ij} + \beta_1 \mathrm{Congruence}_{ij} + \beta_2 \mathrm{Modality}_{ij} + \beta_3 \mathrm{Congruence}_{ij} \times \mathrm{Modality}_{ij} + \beta_4 \mathrm{Category}_{ij} + \beta_5 \mathrm{Gender}_{ij} + \beta_6 \mathrm{Trial}_{ij} + \beta_7 \mathrm{Delay}_{ij} + \beta_8 \mathrm{Similarity}_{ij} + \varepsilon_{ij},$$

$$\varepsilon_{ijk} \sim N(0, \sigma^2(\varepsilon_{ij})),$$

$$\begin{pmatrix} \alpha_i \\ \beta_i \end{pmatrix} \sim N\left(\begin{bmatrix} 0 \\ 0 \end{bmatrix}, \begin{bmatrix} \sigma^2(\alpha_i) & \sigma(\alpha_i, \beta_i) \\ \sigma(\beta_i, \alpha_i) & \sigma^2(\beta_i) \end{bmatrix}\right),$$

$$\alpha_{ij} \sim N(0, \sigma^2(\alpha_{ij}))$$

In simple effects models, the Congruence-by-Modality conditions were dummy coded with predictors either for Visual-congruent, Olfactory-congruent and Visual-incongruent, or for Visual-incongruent, Olfactory-incongruent and Visual-incongruent, e.g.

$$y_{ij} = \alpha_0 + \alpha_i + \alpha_j + \beta_i \mathrm{Trial}_{ij} + \beta_1 \mathrm{VisualCongruent}_{ij} + \beta_2 \mathrm{VisualIncongruent}_{ij} + \beta_3 \mathrm{OlfactoryIncongruent}_{ij} + \beta_4 \mathrm{Category}_{ij} + \beta_5 \mathrm{Gender}_{ij} + \beta_6 \mathrm{Trial}_{ij} + \beta_7 \mathrm{Delay}_{ij} + \beta_8 \mathrm{Similarity}_{ij} + \varepsilon_{ij},$$

$$\varepsilon_{ijk} \sim N(0, \sigma^2(\varepsilon_{ij})),$$

$$\begin{pmatrix} \alpha_i \\ \beta_i \end{pmatrix} \sim N\left(\mu = \begin{bmatrix} 0 \\ 0 \end{bmatrix}, \Sigma = \begin{bmatrix} \sigma^2(\alpha_i) & \sigma(\alpha_i, \beta_i) \\ \sigma(\beta_i, \alpha_i) & \sigma^2(\beta_i) \end{bmatrix}\right),$$

$$\alpha_j \sim N(0, \sigma^2(\alpha_j))$$

All models also included random intercepts for participants and items, and by-participants random slope for Trial number.

In the Bayesian analyses, we used weakly informative priors, as suggested by Stan Development Team (2017), Gelman (2006), and Gelman, Jakulin, Pittau and Su (2008). For error terms $\sigma^2(\varepsilon_{ij})$, $\sigma^2(\alpha_i)$, $\sigma^2(\alpha_j)$ and $\sigma^2(ß_i)$, we used Cauchy(0, 2) priors. For the intercept α_0 and all the fixed effects coefficients β_x, we used $N(0, 1)$. As hyperpriors for the $(\alpha_i, ß_i)$ multivariate distribution, we used $N(0, 1)$ for the mean vector μ, and for the variance-covariance matrix Σ of the $(\alpha_i, ß_i)$ multivariate distribution, we used a LKJ Correlation Distribution with a shape parameter of 2, using Cholesky factorization (see Stan Development Team 2017). Complete model specifications in Stan and R code can be found at osf.io/7qnwu/.

RT data was log-transformed in order to ensure normality. Continuous variables were standardized by subtracting the mean and dividing by two standard deviations. Categorical variables were effect-coded through centering[2]. Trials with RTs below 200ms or above 5s were considered as outliers and excluded from all analyses. In simple effects models, the Congruence-by-Modality conditions were dummy coded with predictors either for Visual-congruent, Olfactory-congruent and Visual-incongruent, or for Visual-incongruent, Olfactory-incongruent and Visual-incongruent.

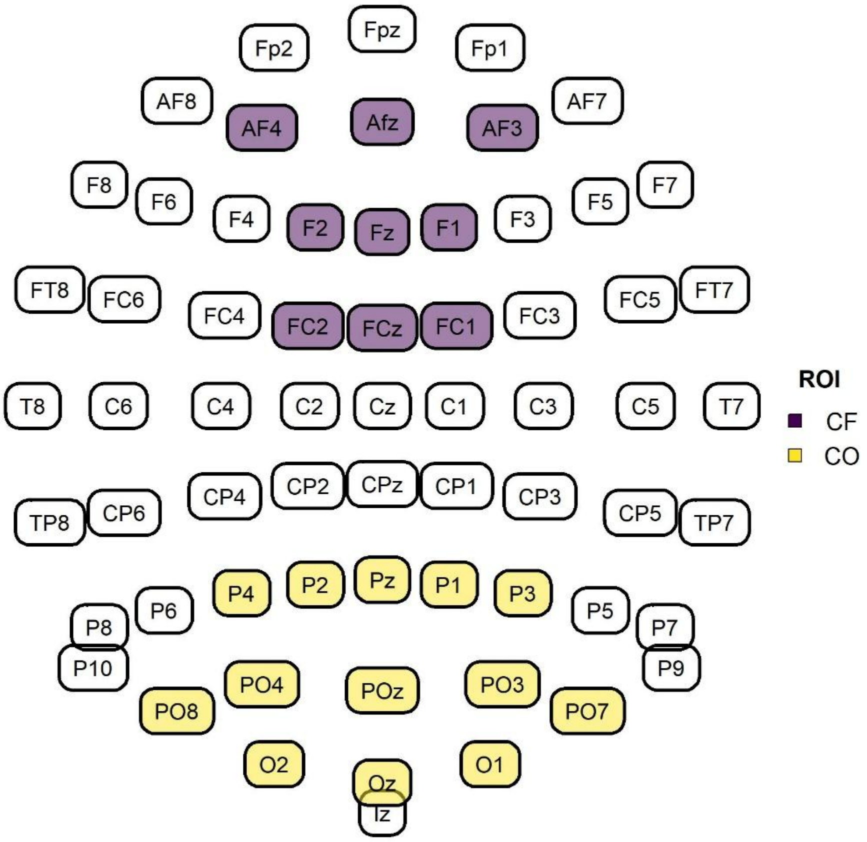

Figure A10. Regions of interest in which mean amplitudes were analyzed.

Analyses of ERP data

In order to identify regions of interest in the spatio-temporal ERP structure, the ERP data was analyzed with a cluster-based permutation test (Maris, 2012; similar too, e.g., Maris & Oostenveld, 2007), as implemented in the ERP analysis toolkit Fishermans' Friend (Hörberg forthcoming). This

[2] That is, by coding the variable as 0 or 1 and subtracting the mean, see e.g. Gelman and Hill (2006: 55).

analysis is presented in more detail in the main manuscript. For the stimulus presentation ERP data, we performed a cluster-based permutation test that compared the congruent and incongruent condition. For auditory target ERPs, we first compared the congruent and incongruent conditions across modalities and then compared modality differences within the congruent and the incongruent conditions seperately. These analyses are presented below.

We also conducted analyses on mean ERP amplitudes across the centro-frontal (CF) scalp region (electrodes AF3, Afz, AF4, F1, Fz, F2, FC1, FCz, FC2), and the centro-occipital (CO) region (electrodes P4, P2, Pz, P1, P3, PO8, PO4, POz, PO3, PO7, O2, Oz, O1). These are illustrated in Figure A10. Time windows were choosen on the basis of previous findings (see above) and on the basis of visual inspection of the data. For stimulus presentation ERPs, we conducted two analyses in the 350-500 ms time window, one within the CF ROI and one in the CO ROI, and two in the 750-1000 ms, again within each ROI. For auditory target ERPs, we conducted two analyses in the 275-300 ms time window within each ROI, and an analysis in the 320-580 ms time window in the CF ROI. In addition to these preregistered analyses, we also conducted analyses in the 600-700, 700-800, and 800-900 ms time windows in the CO ROI that were based upon visual inspection of the data.

Model results

In the following, we report full results of both the Bayesian and the frequentist mixed effects models, together with results of frequentist and Bayesian model comparisons. For simple-effects analyses, we only report effects for the dummy coded simple effect predictors, as the other predictors are identical to those in the initial analyses. We start out with analyses of RTs, followed by analyses of accuracy data, stimulus presentation ERPs, and finally auditory target ERPs.

RT data models

Table A1 shows results of both the Bayesian and Frequentist models of RT data. The two bottom panels show results of the simple effects analyses, testing the effect of Modality in the congruent and the Congruence or Congruence × Modality parameters were significant in the full model, that the

Table A1. Parameter statistics of Bayesian and Frequentist linear mixed effects models of RTs.

**Model**	**Coefficient**	**Bayesian**					**Frequentist**				
		Beta	S. E.	Lower	Upper	*p*	Beta	S.E.	df	t	*p*
**Full model**	(Intercept)	0.17	0.07	0.04	0.30	.009	0.18	0.06	35.56	2.91	.006
	Congruence	0.45	0.03	0.38	0.52	<.0001	0.45	0.03	8.05	15.60	<.0001
	Modality	0.04	0.02	0.00	0.08	.041	0.04	0.02	4321.38	2.01	.045
	Congruence × Modality	0.06	0.03	0.00	0.11	.041	0.06	0.03	4312.08	2.06	.040
	Category	0.02	0.02	-0.02	0.05	.383	0.02	0.02	300.12	0.88	.381
	Sex	0.14	0.13	-0.11	0.40	.280	0.14	0.12	33.00	1.21	.236
	Trial	-0.35	0.03	-0.42	-0.28	<.0001	-0.35	0.03	34.13	-11.40	<.0001
	Delay	0.02	0.01	-0.01	0.04	.229	0.02	0.01	4325.91	1.23	.217
	Similarity	0.01	0.02	-0.03	0.05	.516	0.01	0.02	4300.98	0.64	.524
**Congruent**	VisualCongruent	0.01	0.03	-0.04	0.06	.571	0.01	0.02	4322.20	0.55	.583
	OlfactoryIncongruent	0.43	0.04	0.35	0.50	<.0001	0.43	0.03	12.02	13.24	<.0001
	VisualIncongruent	0.50	0.04	0.41	0.57	<.0001	0.50	0.04	18.33	13.86	<.0001
**Incongruent**	OlfactoryCongruent	-0.42	0.04	-0.50	-0.35	<.0001	-0.50	0.04	18.33	-13.86	<.0001
	VisualCongruent	-0.41	0.04	-0.49	-0.33	<.0001	-0.48	0.03	12.01	-14.99	<.0001
	VisualIncongruent	0.07	0.03	0.02	0.12	.009	0.07	0.03	4322.59	2.80	.005

incongruent conditions separately (see main text). Table A2 shows both Bayesian and Frequentist model statistics (WAIC / AIC, log Likelihood and model deviance) and model comparisons. If either parameter was also tested using model comparison. This was done by comparing the full model to a model in which the parameter at hand was excluded, using Bayes factor (Bayesian models) or the likelihood ratio (frequentist models).

Table A2. Model statistics and null model comparisons of Bayesian and Frequentist linear mixed effects models of RTs.

**Model**		**Bayesian**				**Frequentist**		
		Full model	No Congruence	No interaction		Full model	No Congruence	No interaction
**Full**	WAIC	5619.61	5621.96	5622.64	AIC	5772.89	5806.46	5775.12
	logLik	-2771.37	-2771.41	-2773.29	logLik	-2872.44	-2890.23	-2874.56
	Deviance	5542.74	5542.83	5546.57	Deviance	5744.89	5780.46	5749.12
				Full model vs. No Congruence model				Full model vs. No Congruence model
	BF10			266468.21	$\chi^2(1)$			35.57
	BF01			0	*p*			<.0001
				Full model vs. No interaction model				Full model vs. No interaction model
	BF10			0.22	$\chi^2(1)$			4.23
	BF01			4.5	*p*			.040
**Congruent**		Full model		No Visual-congruent		Full model		No Visual-congruent
	WAIC	5620.41		5618.37	AIC	5771.19		5772.89
	logLik	-2771.59		-2771.32	logLik	-2872.6		-2872.44
	Deviance	5543.19		5542.63	Deviance	5745.19		5744.89
				Full model vs. Visual-congruent model				Full model vs. No Visual-congruent model
	BF10			0.03	$\chi^2(1)$			0.30
	BF01			34.31	*p*			.580
**Incongruent**		Full model		No Visual-incongruent		Full model		No Visual-Incongruent
	WAIC	5619.76		5625.95	AIC	5778.75		5772.89
	logLik	-2771.47		-2774.96	logLik	-2876.37		-2872.44
	Deviance	5542.94		5549.91	Deviance	5752.75		5744.89
				Full model vs. No Visual-incongruent model				Full model vs. No Visual-incongruent model
	BF10			1.45	$\chi^2(1)$			7.86
	BF01			0.69	*p*			.005

Accuracy data models

Table A3 shows results of both the Bayesian and Frequentist models of Accuracy data, the two bottom panels showing results of the simple effects analyses. Table A4 shows both Bayesian and Frequentist model statistics and model comparisons. If either the Congruence or Congruence × Modality parameters were significant in the full model, that parameter was also tested using model comparison, comparing the full model to a model in which the parameter at hand was excluded, using Bayes factor (Bayesian models) or the likelihood ratio (frequentist models).

Table A3. Parameter statistics of Bayesian and Frequentist logistic mixed effects models of Accuracy data.

Model	Coefficient	Bayesian					Frequentist			
		Beta	S. E.	Lower	Upper	*p*	Beta	S.E.	z	*p*
Full model	(Intercept)	3.68	0.26	3.18	4.18	<.0001	3.73	0.23	16.21	<.0001
	Congruence	-1.34	0.33	-1.96	-0.63	.001	-1.55	0.29	-5.38	<.0001
	Modality	0.58	0.24	0.11	1.07	.012	0.66	0.26	2.55	.011
	Congruence × Modality	-0.8	0.35	-1.49	-0.11	.023	-1.00	0.39	-2.57	.010
	Category	0.19	0.17	-0.13	0.52	.252	0.17	0.16	1.05	.295
	Sex	-0.31	0.37	-1.07	0.46	.391	-0.28	0.37	-0.75	.453
	Trial	0.54	0.24	0.07	1.04	.020	0.67	0.26	2.58	.010
	Delay	-0.12	0.15	-0.4	0.18	.426	-0.12	0.14	-0.83	.406
	Similarity	-0.26	0.21	-0.68	0.17	.210	-0.26	0.21	-1.19	.233
Congruent	VisualCongruent	1.13	0.36	0.44	1.83	.001	1.16	0.39	2.98	.003
	OlfactoryIncongruent	-0.85	0.32	-1.44	-0.17	.017	-1.06	0.29	-3.62	<.0001
	VisualIncongruent	-0.66	0.36	-1.33	0.09	.075	-0.90	0.34	-2.61	.009
Incongruent	OlfactoryCongruent	0.81	0.33	0.13	1.44	.022	1.05	0.29	3.62	<.0001
	VisualCongruent	1.82	0.41	0.98	2.62	<.0001	2.21	0.43	5.18	<.0001
	VisualIncongruent	0.12	0.24	-0.35	0.59	.627	0.15	0.24	0.64	.519

Table A4. Model statistics and null model comparisons of Bayesian and Frequentist linear mixed effects models of Accuracy.

**Model**		**Bayesian**				**Frequentist**		
**Full**		Full model	No congruence	No interaction		Full model	No congruence	No interaction
	WAIC	1470.92	1472.56	1474.83	AIC	1514.84	1528.04	1519.62
	logLik	-709.55	-709.37	-711.65	logLik	-744.42	-752.02	-747.81
	Deviance	1419.09	1418.73	1423.3	Deviance	1488.84	1504.04	1495.62
		Full model vs. No congruence model				Full model vs. No congruence model		
	BF10			52.9	$\chi^2(1)$			15.20
	BF01			0.02	*p*			<.0001
		Full model vs. No interaction model				Full model vs. No interaction model		
	BF10			4.29	$\chi^2(1)$			6.78
	BF01			0.23	*p*			.010
**Congruent**		Full model		No Visual-congruent		Full model		No Visual-congruent
	WAIC	1470.27		1478.77	AIC	1522.01		1514.840
	logLik	-709.14		-713.66	logLik	-749.01		-744.420
	Deviance	1418.28		1427.33	Deviance	1498.01		1488.840
		Full model vs. No Visual-congruent model				Full model vs. No Visual-congruent model		
	BF10			72.23	$\chi^2(1)$			9.17
	BF01			0.01	*p*			.002
**Incongruent**		Full model		No Visual-incongruent		Full model		No Visual-incongruent
	WAIC	1471.15		1469.26	AIC	1513.26		1514.84
	logLik	-709.35		-709.11	logLik	-744.63		-744.42
	Deviance	1418.7		1418.23	Deviance	1489.26		1488.84
		Full model vs. No Visual-incongruent model				Full model vs. No Visual-incongruent model		
	BF10			0.31	$\chi^2(1)$			0.42
	BF01			3.23	*p*			.518

Stimulus presentation ERP data models

Table A5 shows results of both the Bayesian and Frequentist models of stimulus presentation ERP data in the N400 (350-500 ms) and LNS (750-1000 ms) time windows. Table A4 shows both Bayesian and Frequentist model statistics and model comparisons, done by comparing the full model to a model in which the parameter at hand was excluded, using Bayes factor (Bayesian models) or the likelihood ratio (frequentist models).

Table A5. Parameter statistics of Bayesian and Frequentist linear mixed effects models of single trial amplitudes of object presentation ERPs on mean amplitudes in the N400 (350-500 ms) and LNS (750-1000 ms) time windows of the CF and the CO ROIs.

T.W.	ROI		Bayesian					Frequentist				
		Coefficient	Beta	S. E.	Lower	Upper	*p*	Beta	S. E.	df	t	*p*
N400	CF	(Intercept)	-1.42	0.23	-1.86	-0.95	<.0001	-1.41	0.24	34.18	-6.00	<.0001
		Congruence	-0.37	0.26	-0.89	0.15	.135	-0.40	0.24	7.09	-1.68	.136
		Category	0.10	0.21	-0.31	0.51	.605	0.09	0.21	8.95	0.42	.681
		Congruence × Type	0.31	0.41	-0.48	1.12	.428	0.36	0.42	9.04	0.87	.405
		Sex	-0.27	0.38	-1.01	0.49	.482	-0.32	0.39	33.08	-0.80	.427
		Trial	0.16	0.17	-0.17	0.48	.313	0.17	0.17	34.16	0.98	.335
		Similarity	-0.02	0.12	-0.26	0.21	.844	-0.02	0.12	3716.60	-0.20	.839
	CO	(Intercept)	2.63	0.29	2.04	3.19	<.0001	2.68	0.28	35.70	9.66	<.0001
		Congruence	0.19	0.29	-0.39	0.79	.520	0.18	0.28	7.64	0.64	.540
		Category	0.05	0.24	-0.44	0.50	.819	0.11	0.24	9.22	0.44	.669
		Congruence × Type	-0.57	0.45	-1.44	0.31	.202	-0.73	0.48	9.31	-1.50	.167
		Sex	0.45	0.46	-0.46	1.35	.325	0.55	0.50	33.04	1.12	.272
		Trial	-0.30	0.18	-0.65	0.06	.098	-0.31	0.18	33.19	-1.70	.098
		Similarity	0.05	0.13	-0.19	0.30	.667	0.06	0.13	3691.04	0.44	.662
LNS	CF	(Intercept)	0.72	0.33	0.07	1.33	.027	0.75	0.31	33.70	2.43	.020
		Congruence	-0.60	0.20	-0.99	-0.19	.011	-0.63	0.18	6.11	-3.60	.011
		Category	-0.02	0.18	-0.35	0.34	.862	-0.05	0.16	10.31	-0.32	.758
		Congruence × Category	0.89	0.35	0.19	1.54	.020	1.03	0.33	10.54	3.13	.010
		Sex	-0.10	0.52	-1.17	0.89	.844	-0.15	0.60	32.79	-0.24	.809
		Trial	-0.19	0.18	-0.55	0.16	.281	-0.19	0.18	35.06	-1.05	.299
		Similarity	0.01	0.13	-0.25	0.27	.947	0.01	0.14	3657.29	0.09	.930
	CO	(Intercept)	0.13	0.38	-0.62	0.87	.720	0.11	0.37	35.52	0.29	.776
		Congruence	0.66	0.25	0.17	1.14	.019	0.70	0.23	7.15	3.04	.018
		Category	0.30	0.22	-0.16	0.71	.172	0.36	0.20	10.05	1.76	.109
		Congruence × Category	-0.83	0.42	-1.61	0.05	.058	-1.01	0.41	10.20	-2.47	.033
		Sex	0.23	0.58	-0.91	1.40	.696	0.35	0.73	33.04	0.48	.635
		Trial	0.10	0.20	-0.31	0.50	.597	0.11	0.21	34.35	0.50	.618
		Similarity	-0.10	0.14	-0.37	0.19	.476	-0.10	0.14	3676.79	-0.69	.490

Table A6. Model statistics and null model comparisons of Bayesian and Frequentist linear mixed effects models of single trial amplitudes of object presentation ERPs on mean amplitudes in the N400 (350-500 ms) and LNS (750-1000 ms) time windows of the CF and the CO ROIs.

**T.W.**	**ROI**		**Bayesian**			**Frequentist**	
			Full model	No congruence		Full model	No congruence
**N400**	CF	WAIC	20787.97	20787.02	AIC	20852.86	20854.04
		logLik	-10365.82	-10365.23	logLik	-10414.43	-10416.02
		Deviance	20731.63	20730.47	Deviance	20828.86	20832.04
			Full model vs. No congruence			Full model vs. No congruence	
		BF10		0.79	$\chi^2(1)$		3.18
		BF01		1.26	*p*		.074
	CO		Full model	No congruence		Full model	No congruence
		WAIC	21017.56	21017.43	AIC	21094.87	21093.39
		logLik	-10479.80	-10479.71	logLik	-10535.43	-10535.70
		Deviance	20959.6	20959.41	Deviance	21070.87	21071.39
			Full model vs. No congruence			Full model vs. No congruence	
		BF10		0.42	$\chi^2(1)$		0.52
		BF01		2.41	*p*		.469
**LNS**	CF		Full model	No congruence		Full model	No congruence
		WAIC	21679.97	21681.89	AIC	21756.45	21764.22
		logLik	-10814.03	-10813.82	logLik	-10866.23	-10871.11
		Deviance	21628.07	21627.65	Deviance	21732.45	21742.22
			Full model vs. No congruence			Full model vs. No congruence	
		BF10		10.46	$\chi^2(1)$		9.77
		BF01		0.1	*p*		.002
	CO		Full model	No congruence		Full model	No congruence
		WAIC	21765.46	21765.02	AIC	21856.37	21862.22
		logLik	-10852.67	-10852.02	logLik	-10916.18	-10920.11
		Deviance	21705.35	21704.04	Deviance	21832.37	21840.22
			Full model vs. No congruence			Full model vs. No congruence	
		BF10		4.71	$\chi^2(1)$		7.85
		BF01		0.21	*p*		.005

Auditory target ERP data models

Table A7 shows results of Bayesian and Frequentist models of auditory target ERP data in the N2 (275-300 ms) and the P300 (320-580 ms) time windows. Table A8 shows model statistics and model comparisons for these models, comparing the full models to models in which the parameter at hand is excluded, using Bayes factor (Bayesian models) or the likelihood ratio (Frequentist models).

Table A7. Parameter statistics of Bayesian and Frequentist linear mixed effects models of single trial amplitudes of auditory target ERPs on mean amplitudes in the N2 (275-300 ms) and P300 (320-580 ms) time windows of the CF and the CO ROIs.

**T.W.**	**ROI**	**Coefficient**	**Bayesian**					**Frequentist**				
			Beta	S. E.	Lower	Upper	*p*	Beta	S. E.	df	t	*p*
**N2**	CF	(Intercept)	-3.89	0.29	-4.46	-3.32	<.0001	-3.94	0.28	30.26	-14.10	<.0001
		Congruence	0.26	0.16	-0.06	0.57	.094	0.27	0.14	3374.80	1.93	.054
		Modality	0.13	0.21	-0.29	0.53	.530	0.14	0.22	3377.72	0.64	.524
		Congruence × Modality	0.12	0.27	-0.41	0.65	.665	0.13	0.28	3372.86	0.48	.629
		Category	0.24	0.15	-0.06	0.53	.112	0.25	0.14	3376.07	1.84	.066
		Sex	-0.32	0.48	-1.23	0.64	.491	-0.47	0.52	30.03	-0.91	.370
		Trial	0.06	0.19	-0.31	0.42	.732	0.07	0.19	32.82	0.38	.708
		Delay	0.05	0.14	-0.20	0.33	.733	0.05	0.14	3395.21	0.37	.709
		Similarity	0.08	0.20	-0.31	0.49	.682	0.10	0.21	3377.33	0.49	.626
	CO	(Intercept)	4.01	0.32	3.37	4.66	<.0001	4.12	0.32	30.13	13.01	<.0001
		Congruence	-0.09	0.17	-0.41	0.24	.564	-0.09	0.14	3351.61	-0.67	.501
		Modality	-0.23	0.21	-0.65	0.19	.284	-0.25	0.22	3355.82	-1.12	.262
		Congruence × Modality	-0.12	0.26	-0.63	0.38	.652	-0.14	0.28	3349.38	-0.51	.607
		Category	-0.33	0.15	-0.62	-0.03	.028	-0.34	0.14	3352.27	-2.43	.015
		Sex	0.59	0.52	-0.45	1.58	.238	0.78	0.58	30.23	1.34	.190
		Trial	-0.06	0.22	-0.51	0.38	.789	-0.08	0.22	31.48	-0.37	.716
		Delay	0.01	0.14	-0.26	0.29	.916	0.01	0.14	3369.29	0.10	.920
		Similarity	-0.26	0.20	-0.65	0.13	.201	-0.28	0.21	3355.46	-1.35	.179
**P300**	CF	(Intercept)	-2.27	0.32	-2.89	-1.63	<.0001	-2.31	0.32	30.43	-7.30	<.0001
		Congruence	0.71	0.15	0.40	1.01	.001	0.73	0.14	8.33	5.31	.001
		Modality	0.14	0.18	-0.20	0.50	.433	0.15	0.19	3328.67	0.81	.419
		Congruence × Modality	0.13	0.23	-0.31	0.56	.605	0.13	0.24	3395.20	0.56	.578
		Category	0.17	0.13	-0.08	0.44	.172	0.18	0.13	34.13	1.37	.179
		Sex	-0.49	0.53	-1.54	0.55	.347	-0.70	0.62	29.82	-1.12	.271
		Trial	-0.16	0.14	-0.43	0.11	.261	-0.16	0.14	706.05	-1.16	.247
		Delay	0.01	0.12	-0.21	0.24	.930	0.01	0.12	3401.21	0.06	.952
		Similarity	-0.05	0.17	-0.38	0.30	.771	-0.05	0.18	3135.00	-0.26	.792

Table A8. Model statistics and null model comparisons of Bayesian and Frequentist linear mixed effects models of single trial amplitudes of auditory target ERPs on mean amplitudes in the N2 (275-300 ms) and P300 (320-580 ms) time windows of the CF and the CO ROIs.

**T.W.**	**ROI**		**Bayesian**			**Frequentist**	
			Full model	No congruence		Full model	No congruence
**N2**	CF	WAIC	19412.41	19415.58	AIC	19476.06	19477.78
		logLik	-9681.21	-9682.37	logLik	-9724.03	-9725.89
		Deviance	19362.42	19364.74	Deviance	19448.06	19451.78
			Full model vs. No congruence model			Full model vs. No congruence model	
		BF10		0.72	$\chi^2(1)$		3.73
		BF01		1.39	*p*		.053
	CO		Full model	No congruence		Full model	No congruence
		WAIC	19258.06	19256.94	AIC	19332.80	19331.26
		logLik	-9601.13	-9601.00	logLik	-9652.40	-9652.63
		Deviance	19202.26	19202.00	Deviance	19304.80	19305.26
			Full model vs. No congruence model			Full model vs. No congruence model	
		BF10		0.14	$\chi^2(1)$		0.45
		BF01		7.28	*p*		.500
**P300**	CF		Full model	No congruence model		Full model	No congruence model
		WAIC	18352.57	18358.04	AIC	18435.72	18448.42
		logLik	-9153.28	-9153.62	logLik	-9203.86	-9211.21
		Deviance	18306.57	18307.24	Deviance	18407.72	18422.42
			Full model vs. No congruence model			Full model vs. No congruence model	
		BF10		105.20	$\chi^2(1)$		14.70
		BF01		0.01	*p*		<.001

Table A9 shows results of Bayesian and Frequentist models of auditory target ERP data in the LPC1 (600-700 ms), LPC2 (700-800 ms), and LPC3 (800-900 ms) time windows, together with results of simple effects analyses. Tables A9-A11 show model statistics and model comparisons for these models, comparing the full models to models in which the parameter at hand is excluded, using Bayes factor (Bayesian models) or the likelihood ratio (Frequentist models). If either the Congruence, Modality or Congruence × Modality parameters were significant in any of the full models, that parameter was tested with model comparison.

Table A9. Parameter statistics of Bayesian and Frequentist linear mixed effects models of single trial amplitudes of auditory target ERPs on mean amplitudes in the LPC1 (600-700 ms), LPC2 (700-800 ms) and the LPC3 (800-900 ms) time windows of the CO ROI.

**T.W.**	**Coefficient**	**Bayesian**					**Frequentist**				
		Beta	S.E.	Lower	Upper	*p*	Beta	S.E.	df	t	*p*
**LPC1**	(Intercept)	2.41	0.32	1.76	3.02	<.0001	2.48	0.30	29.96	8.27	<.0001
	Congruence	0.66	0.17	0.31	1.00	<.0001	0.68	0.15	3361.99	4.58	<.0001
	Modality	0.14	0.22	-0.29	0.58	.549	0.10	0.23	3372.57	0.43	.668
	Congruence × Modality	0.22	0.29	-0.34	0.78	.458	0.26	0.29	3360.93	0.90	.369
	Category	-0.34	0.16	-0.65	-0.03	.030	-0.35	0.15	3362.81	-2.37	.018
	Sex	0.40	0.51	-0.65	1.37	.434	0.55	0.58	29.93	0.94	.354
	Trial	0.01	0.19	-0.35	0.38	.941	0.00	0.18	31.11	0.03	.980
	Delay	-0.25	0.14	-0.52	0.02	.083	-0.26	0.15	3383.42	-1.74	.082
	Similarity	0.17	0.22	-0.26	0.59	.438	0.13	0.22	3372.29	0.58	.562
	VisualCongruent	-0.01	0.27	-0.54	0.53	.949	-0.03	0.28	3368.90	-0.12	.902
	OlfactoryIncongruent	0.53	0.22	0.09	0.96	.024	0.54	0.21	3363.83	2.62	.009
	VisualIncongruent	0.76	0.28	0.21	1.31	.009	0.78	0.27	3366.85	2.82	.005
	OlfactoryCongruent	-0.51	0.22	-0.94	-0.07	.024	-0.54	0.21	3363.83	-2.62	.009
	VisualCongruent	-0.49	0.27	-1.03	0.03	.064	-0.58	0.27	3372.65	-2.11	.035
	VisualIncongruent	0.30	0.26	-0.21	0.80	.262	0.23	0.27	3370.40	0.84	.399
**LPC2**	(Intercept)	2.13	0.35	1.52	2.81	<.0001	2.24	0.31	30.29	7.11	<.0001
	Congruence	0.54	0.20	0.15	0.93	.014	0.57	0.17	7.91	3.31	.011
	Modality	0.26	0.22	-0.17	0.69	.245	0.27	0.24	3274.50	1.16	.248
	Congruence × Modality	0.55	0.27	0.03	1.07	.039	0.59	0.30	3352.86	1.97	.049
	Category	-0.38	0.16	-0.71	-0.05	.026	-0.40	0.16	30.25	-2.44	.021
	Sex	0.40	0.52	-0.62	1.44	.530	0.60	0.62	29.88	0.96	.347
	Trial	-0.02	0.19	-0.40	0.34	.936	-0.04	0.19	27.39	-0.19	.851
	Delay	-0.17	0.14	-0.46	0.11	.225	-0.17	0.15	3383.32	-1.11	.269
	Similarity	0.06	0.21	-0.35	0.47	.859	0.07	0.23	3042.24	0.30	.761
	VisualCongruent	-0.06	0.26	-0.59	0.45	.809	-0.02	0.28	3325.24	-0.09	.930
	OlfactoryIncongruent	0.25	0.24	-0.21	0.73	.298	0.27	0.23	24.39	1.20	.242
	VisualIncongruent	0.80	0.30	0.21	1.39	.010	0.84	0.29	63.67	2.88	.005
	OlfactoryCongruent	-0.24	0.24	-0.72	0.25	.310	-0.27	0.23	24.39	-1.20	.242
	VisualCongruent	-0.28	0.29	-0.84	0.29	.328	-0.30	0.29	63.04	-1.02	.312
	VisualIncongruent	0.57	0.27	0.05	1.08	.033	0.57	0.28	3326.75	2.04	.042
**LPC3**	(Intercept)	0.97	0.32	0.35	1.60	.003	1.00	0.30	29.76	3.27	.003
	Congruence	0.28	0.19	-0.10	0.63	.137	0.29	0.16	3366.17	1.87	.062
	Modality	0.22	0.23	-0.23	0.66	.338	0.24	0.24	3370.61	0.98	.328
	Congruence × Modality	0.77	0.30	0.17	1.35	.012	0.84	0.31	3364.44	2.71	.007
	Category	-0.39	0.16	-0.71	-0.09	.011	-0.40	0.16	3367.29	-2.56	.010
	Sex	0.22	0.54	-0.86	1.27	.668	0.32	0.61	29.76	0.53	.603
	Trial	0.19	0.20	-0.22	0.59	.346	0.19	0.21	29.84	0.88	.389
	Delay	-0.18	0.15	-0.48	0.12	.244	-0.18	0.16	3386.30	-1.16	.247
	Similarity	0.00	0.22	-0.45	0.43	.989	0.00	0.23	3370.43	0.01	.991
	VisualCongruent	-0.19	0.27	-0.71	0.33	.480	-0.18	0.29	3368.60	-0.64	.524
	OlfactoryIncongruent	-0.13	0.23	-0.58	0.32	.575	-0.13	0.22	3366.73	-0.58	.561
	VisualIncongruent	0.50	0.28	-0.03	1.04	.072	0.53	0.29	3365.97	1.84	.067
	OlfactoryCongruent	0.11	0.23	-0.34	0.58	.660	0.13	0.22	3366.73	0.58	.561
	VisualCongruent	-0.09	0.28	-0.62	0.45	.757	-0.06	0.29	3372.50	-0.20	.843
	VisualIncongruent	0.60	0.27	0.07	1.13	.032	0.66	0.29	3369.19	2.28	.023

Table A10. Model statistics and null model comparisons of Bayesian and Frequentist linear mixed effects models of single trial amplitudes of auditory target ERPs on mean amplitudes in the LPC1 (600-700 ms) time window in the CO ROI.

	**Bayesian**			**Frequentist**	
	Full model	No congruence		Full model	No congruence
WAIC	19764.43	19771.69	AIC	19828.97	19839.82
logLik	-9858.26	-9859.72	logLik	-9900.49	-9906.91
Deviance	19716.52	19719.44	Deviance	19800.97	19813.82
	Full model vs. No congruence model			Full model vs. No congruence model	
BF10		40.23	$\chi^2(1)$		12.84
BF01		0.02	*p*		.0003

Table A11. Model statistics and null model comparisons of Bayesian and Frequentist linear mixed effects models of single trial amplitudes of auditory target ERPs on mean amplitudes in the LPC2 (700-800 ms) time window in the CO ROI.

**Model**		**Bayesian**				**Frequentist**		
**Full**		Full model	No congruence	No interaction		Full model	No congruence	No interaction
	WAIC	19915.96	19918.72	19918.32	AIC	19983.09	19989.52	19985.20
	logLik	-9933.77	-9933.86	-9935.36	logLik	-9977.55	-9981.76	-9979.60
	Deviance	19867.53	19867.72	19870.71	Deviance	19955.09	19963.52	19959.20
		Full model vs. No congruence model				Full model vs. No congruence model		
	BF10			8.71	$\chi^2(1)$			8.42
	BF01			0.11	*p*			.003
		Full model vs. No interaction model				Full model vs. No interaction model		
	BF10			2.95	$\chi^2(1)$			4.1
	BF01			0.34	*p*			.042
**Congruent**		Full model		No Visual-congruent		Full model		No Visual-congruent
	WAIC	19915.65		19913.53	AIC	19983.09		19981.11
	logLik	-9933.5		-9932.81	logLik	-9977.55		-9977.56
	Deviance	19867		19865.61	Deviance	19955.09		19955.11
		Full model vs. No Visual-congruent model				Full model vs. No Visual-congruent model		
	BF10			0.24	$\chi^2(1)$			0.02
	BF01			4.08	*p*			.885
**Incongruent**		Full model		No Visual-incongruent		Full model		No Visual-incongruent
	WAIC	19915.65		19918.45	AIC	19983.09		19985.25
	logLik	-9933.39		-9935.40	logLik	-9977.55		-9979.62
	Deviance	19866.77		19870.79	Deviance	19955.09		19959.25
		Full model vs. No Visual-incongruent model				Full model vs. No Visual-incongruent model		
	BF10			2.7	$\chi^2(1)$			4.16
	BF01			0.37	*p*			.041

Table A12. Model statistics and null model comparisons of Bayesian and Frequentist linear mixed effects models of single trial amplitudes of auditory target ERPs on mean amplitudes in the LPC3 (800-900 ms) time window in the CO ROI.

**Model**		**Bayesian**			**Frequentist**	
		Full model	No interaction		Full model	No interaction
	WAIC	20143.55	20148.57	AIC	20207.1	20212.44
	logLik	-10047.26	-10050.14	logLik	-10089.55	-10093.22
**Full**	Deviance	20094.52	20100.29	Deviance	20179.1	20186.44
		Full model vs. No interaction model			Full model vs. No interaction model	
	BF10		7.47	$\chi^2(1)$		7.34
	BF01		0.13	*p*		.006
		Full model	No Visual-congruent		Full model	No Visual-congruent
	WAIC	20142.2	20142.03	AIC	20207.1	20205.51
	logLik	-10046.57	-10046.76	logLik	-10089.55	-10089.76
**Congruent**	Deviance	20093.14	20093.52	Deviance	20179.1	20179.51
		Full model vs. No Visual-congruent model			Full model vs. No Visual-congruent model	
	BF10		0.37	$\chi^2(1)$		0.41
	BF01		2.67	*p*		.524
		Full model	No Visual-incongruent		Full model	No Visual-incongruent
	WAIC	20142.64	20146.7	AIC	20207.1	20210.33
	logLik	-10046.69	-10049.14	logLik	-10089.55	-10092.16
**Incongruent**	Deviance	20093.39	20098.28	Deviance	20179.1	20184.33
		Full model vs. No Visual-incongruent model			Full model vs. No Visual-incongruent model	
	BF10		4.98	$\chi^2(1)$		5.22
	BF01		0.2	*p*		.022

Stimulus presentation ERP effects

Cluster-based permutation analysis

The cluster-based permutation analysis of the stimulus presentation ERPs identified one negative cluster with a centro-frontal and centro-parietal scalp distribution, spanning the 750-1000 ms time window, and one positive cluster with a centro-parietal and centro-occipital scalp distribution in the 750-1000 ms time window. These clusters are illustrated in Figure A11.

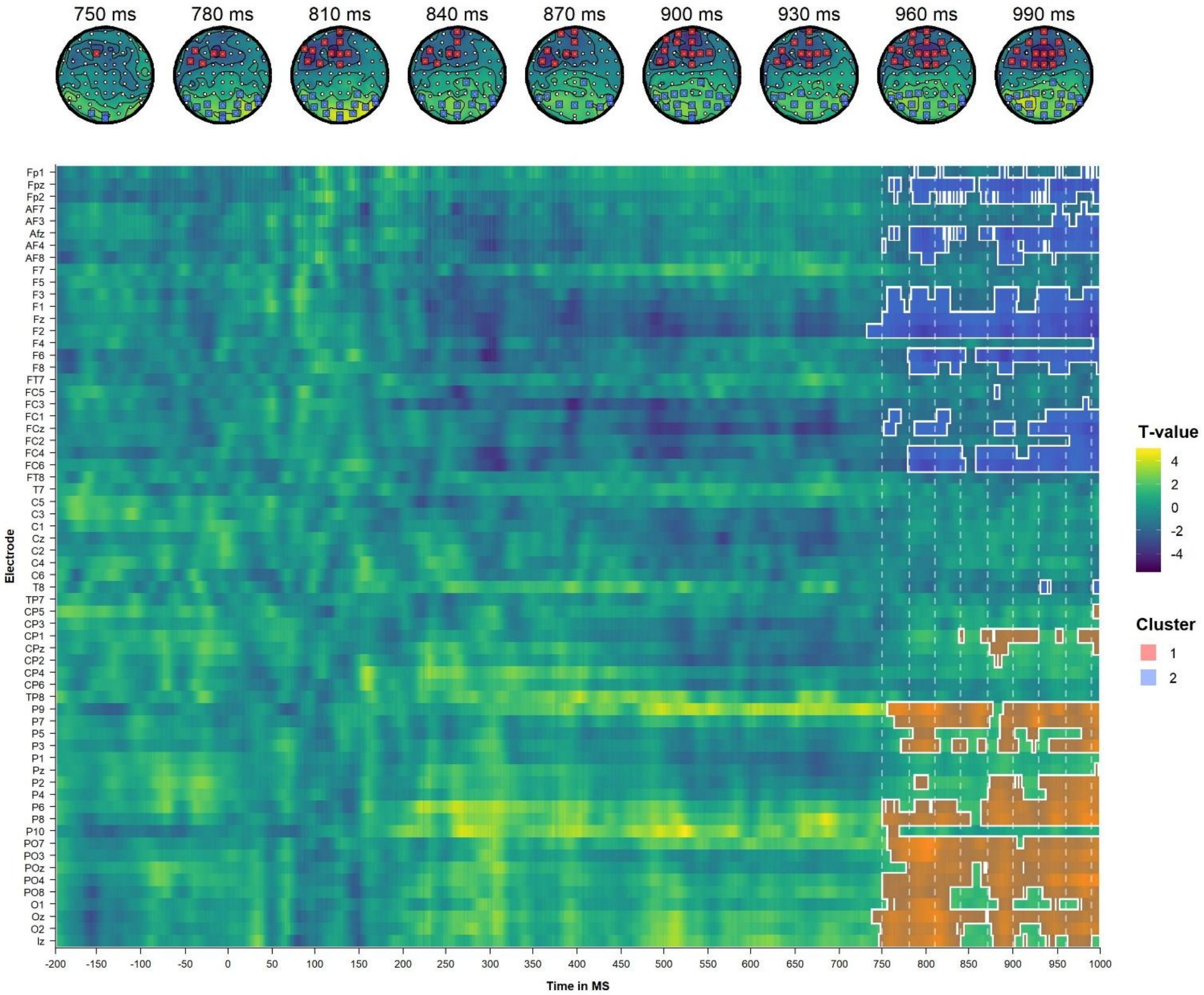

Figure A11. Results of the cluster-based permutation analysis of the stimulus presentation ERPs. The top panel illustrates the scalp topographies of the identified clusters (ordered by size) at selected time points. The bottom panel illustrates the spatio-temporal distribution of the identified clusters. The dashed lines correspond to the selected time points of the topoplots in the upper panel.

Mean ERP analyses

Grand average stimulus presentation ERP:s across each ROI, together with topoplots of Congurent-Incongruent grand average differences in the 350-500 and 750-1000 ms time windows are shown in Figure A12. The figure indicates that incongruent trials engender a dipolar scalp effect, ranging from 300 ms to the end of the epoch, which is negative in the frontal region and positive in the parietal-occipital region. As shown in Table A6, the analyses in the 350-500 ms time window did not find a Congruence effect neither in the CF ROI, β = -0.37, S.E. = 0.26, CI = [-0.89, 0.15], p = .135, nor in the CO ROI, β = 0.19, S.E. = 0.29, CI = [-0.39, 0.79], p = .520. We also conducted additional analyses in the 750-1000 ms time window. These found a significant negative effect in the CF ROI, β = -0.60,

S.E. = 0.20, CI = [-0.99, -0.19], p = .011, mirrored by a positive effect in the CO ROI, β = 0.66, S.E. = 0.25, CI = [0.17, 1.14], p = .019. Thus, although we did not find strong enough evidence for a congruence-based N400-like effect, contrary to our predictions, incongruent trials engendered a late, dipolar slow wave that is negative in the anterior scalp region and positive in the posterior scalp region. A plausible interpretation of this effect is that it reflects differences in working memory load between congruent and incongruent trials. As illustrated in Figure A1, participants needed to encode and retain information about the stimuli in working memory until the presentation of the auditory target. It is therefore possible that the late, dipolar slow wave is related to differences in working memory load. In the congruent trials, participants only need to retain the category of the multisensory object in working memory, whereas in the incongruent trials, they are required to retain the incongruent olfactory and visual object representations simultaneously. In line with this, several studies investigating ERP correlates to working memory load during retention have found high versus low load to be reflected in slow wave activity (Bosch, Mecklinger and Friederici 2001; Honda et al. 1996; Mecklinger and Pfeifer 1996; Monfort and Pouthas 2003; Ruchkin et al. 1997; Ruchkin, Johnson, Canoune and Ritter 1990). For instance, Rutchkin et al. (1990) found high working memory load during retention to be reflected by a centro-parietal positive slow wave that was followed by frontal negative slow wave (see also Honda et al. 1996), very similar to the effects observed in the present study. It therefore seems plausible that the observed late, dipolar slow wave stems from differences in working memory load.

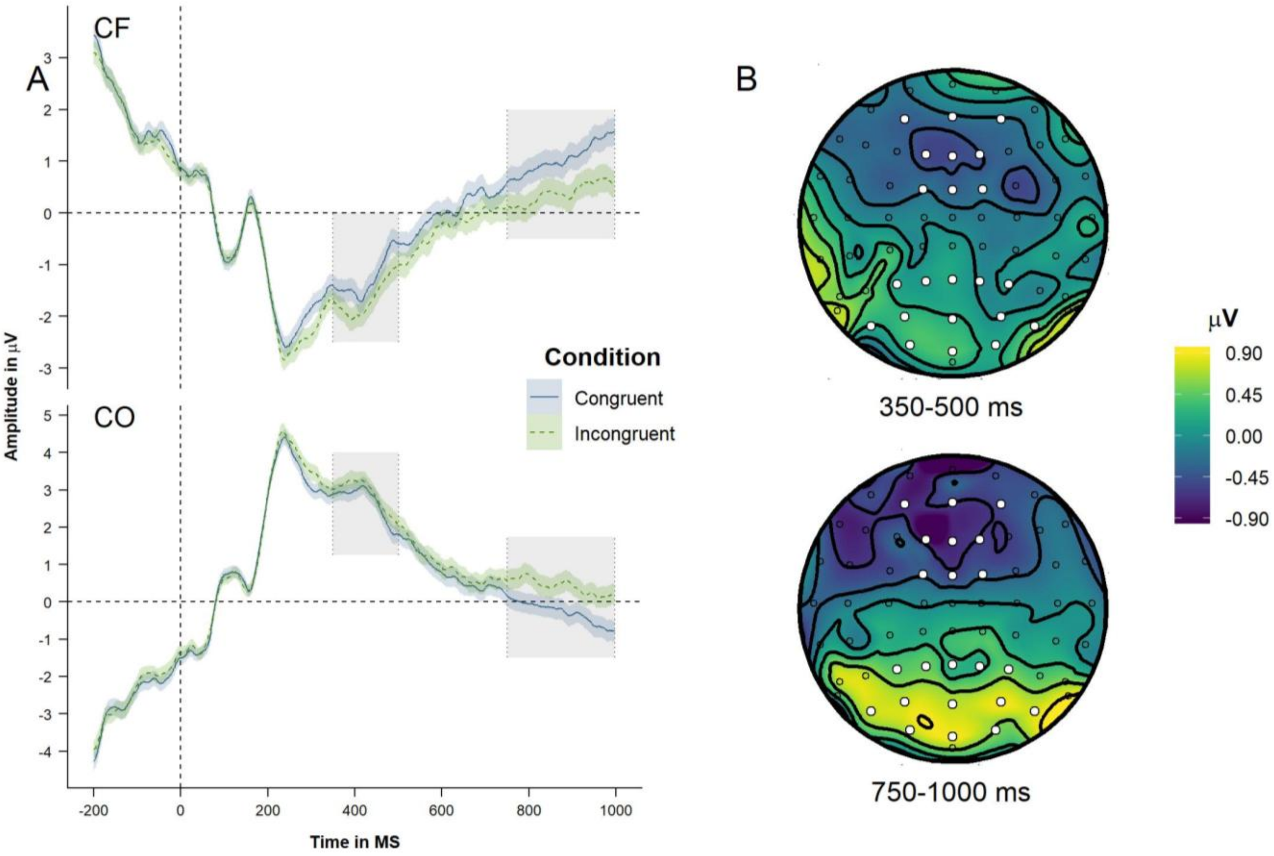

Figure A12. Panel A. Grand average ERPs time-locked to the onset of the visual stimuli, averaged across the CF and CO ROIs. Shaded areas show 95% confidence intervals. Grey areas mark the 350-500 and 750-1000 ms time windows. Panel B. Topographies of Congruent-Incongruent grand average differences in the 350-500 and 750-1000 ms time windows. The positions of the electrodes of each ROI are highlighted in white.

References

Alexander JE, Porjesz B, Bauer LO, Kuperman S, Morzorati S, O'Connor SJ, Rohrbaugh J, Begleiter H, Polich J. 1995. P300 hemispheric amplitude asymmetries from a visual oddball task. Psychophysiology. 32: 467–475.

Amano K, Goda N, Nishida S, Ejima Y, Takeda T, Ohtani Y. 2006. Estimation of the Timing of Human Visual Perception from Magnetoencephalography. J Neurosci. 26: 3981–3991.

Bartholow BD, Pearson MA, Dickter CL, Sher KJ, Fabiani M, Gratton G. 2005. Strategic control and medial frontal negativity: Beyond errors and response conflict. Psychophysiology. 42: 33–42.

Bartlett MS. 1957. A comment on D. V. Lindley's statistical paradox. Biometrika. 44: 533–534.

Bates D, Maechler M, Bolker B, Walker S. 2014. lme4: Linear mixed-effects models using Eigen and S4 (Version R package version 1.1-7). Retrieved from http://CRAN.R-project.org/package=lme4.

Benjamini Y, Hochberg Y. 1995. Controlling the False Discovery Rate: A Practical and Powerful Approach to Multiple Testing. J R Stat Soc B Met. 57: 289–300.

Berchicci M, Spinelli D, Di Russo F. 2016. New insights into old waves. Matching stimulus- and response-locked ERPs on the same time-window. Biol Psychol. 117: 202–215.

Bosch V, Mecklinger A, Friederici AD. 2001. Slow cortical potentials during retention of object, spatial, and verbal information. Cognitive Brain Res. 10: 219–237.

Castle PC, Van Toller S, Milligan GJ. 2000. The effect of odour priming on cortical EEG and visual ERP responses. Int J Psychophysiol. 36: 123–131.

Chen A, Li H, Qiu J, Luo Y. 2006. The time course of visual categorization: Electrophysiological evidence from ERP. Chinese Sci Bull. 51: 1586–1592.

Collins LF, Long CJ. 1996. Visual reaction time and its relationship to neuropsychological test performance. Arch Clin Neuropsych. 11: 613–623.

Coulson S, King JW, Kutas M. 1998. Expect the unexpected: Event-related brain response to morphosyntactic violations. Lang Cognitive Proc. 13: 21–58.

Desmedt JE. 1980. P300 in serial tasks: an essential post-decision closure mechanism. Prog Brain Res. 54: 682–686.

Donchin E. 1981. Surprise!? Surprise? Psychophysiology. 18: 493–513.

Donchin E, Coles MG. 1988. Is the P300 component a manifestation of context updating? Behav Brain Sci. 11(3): 357–427.

Dong G, Yang L, Hu Y, Jiang Y. 2009. Is N2 associated with successful suppression of behavior responses in impulse control processes? NeuroReport. 20: 537–542.

Dunnett CW. 1955. A Multiple Comparison Procedure for Comparing Several Treatments with a Control. J Am Stat Assoc. 50: 1096–1121.

Folstein JR, Van Petten C. 2007. Influence of cognitive control and mismatch on the N2 component of the ERP: A review. Psychophysiology, 45: 152-170.

Gallagher A, Béland R, Vannasing P, Bringas ML, Sosa PV, Trujillo-Barreto NJ, Conolly J, Lassonde M. 2014. Dissociation of the N400 component between linguistic and non-linguistic processing: A source analysis study. World J Neurosci. 4: 25–39.

Geisler MW, Polich J. 1994. P300 habituation from visual stimuli? Physiol Behav. 56(3): 511–516.

Gelman A. 2006. Prior distributions for variance parameters in hierarchical models (comment on article by Browne and Draper). Bayesian Anal. 1: 515–534.

Gelman A, Jakulin A, Pittau MG, Su YS. 2008. A weakly informative default prior distribution for logistic and other regression models. Ann Appl Stat. 2: 1360–1383.

Gelman A, Robert CP. 2013. "Not Only Defended But Also Applied": The Perceived Absurdity of Bayesian Inference. Am Stat. 67: 1–5.

Gelman A, Rubin DB. 1995. Avoiding Model Selection in Bayesian Social Research. Sociol Methodol. 25: 165.

Grigor J, Van Toller S, Behan J, Richardson A. 1999. The effect of odour priming on long latency visual evoked potentials of matching and mismatching objects. Chem Senses. 24: 137–144.

Heil M, Osman A, Wiegelmann J, Rolke B, Hennighausen, E. 2000. N200 in the Eriksen-Task: Inhibitory Executive Processes? J Psychophysiol. 14: 218–225.

Hochberg Y. 1988. A sharper Bonferroni procedure for multiple tests of significance. Biometrika. 75: 800–802.

Honda M, Barrett G, Yoshimura N, Ikeda A, Nagamine T, Shibasaki H. 1996. Event-related potentials during paired associate memory paradigm. Electroen Clin Neuro. 100: 407–421.

Hörberg T. forthcoming. Fishermans Friend: An R-based tool for statistical analysis and visualization of high-density EEG-data.

Hörberg T, Koptjevskaja-Tamm M, Kallioinen P. 2013. The neurophysiological correlate to grammatical function reanalysis in Swedish. Lang Cognitive Proc. 28: 388–416.

Jeffreys H. 1961. Theory of probability. 3rd ed. Oxford (NY): Oxford University Press.

Keidel A, Hörberg T. 2017. Qualitative differences in L3 learners' neurophysiological response to L1 versus L2 transfer. Paper presented at Interspeech 2017, Stockholm.

Kok A. 2001. On the utility of P3 amplitude as a measure of processing capacity. Psychophysiology. 38: 557–577.

Kovic V, Plunkett K, Westermann G. 2009. Shared and/or separate representations of animate/inanimate categories: An ERP study. Psihologija. 42: 5–26.

Kovic V, Plunkett K, Westermann G. 2010. A unitary account of conceptual representations of animate/inanimate categories. Psihologija. 43: 155–165.

Kowalewski J, Murphy C. 2012. Olfactory ERPs in an odor/visual congruency task differentiate ApoE ε4 carriers from non-carriers. Brain Res. 1442: 55–65.

Kutas M, Federmeier KD. 2011. Thirty Years and Counting: Finding Meaning in the N400 Component of the Event-Related Brain Potential (ERP). Annu Rev Psychol. 62: 621–647.

Kutas M, Hillyard SA. 1980. Reading senseless sentences: Brain potentials reflect semantic incongruity. Science. 207: 203–205.

Kutas M, McCarthy G, Donchin E. 1977. Augmenting mental chronometry: the P300 as a measure of stimulus evaluation time. Science. 197: 792–795.

Kuznetsova A, Brockhoff PB, Christensen RH. 2014. lmerTest: Tests for random and fixed effects for linear mixed effect models (lmer objects of lme4 package). Retrieved from http://CRAN.R-project.org/package=lmerTest.

Liotti M, Woldorff MG, Perez R, Mayberg HS. 2000. An ERP study of the temporal course of the Stroop color-word interference effect. Neuropsychologia. 38: 701–711.

Liu B, Wang Z, Jin Z. 2009. The integration processing of the visual and auditory information in videos of real-world events: An ERP study. Neurosci Lett. 461: 7–11.

Maris E. 2012. Statistical testing in electrophysiological studies. Psychophysiology. 49(4): 549–565.

Maris E, Oostenveld R. 2007. Nonparametric statistical testing of EEG- and MEG-data. J Neurosci Meth. 164: 177–190.

Markela-Lerenc J, Ille N, Kaiser S, Fiedler P, Mundt C, Weisbrod M. 2004. Prefrontal-cingulate activation during executive control: which comes first? Cognitive Brain Res. 18: 278–287.

McElreath R. 2016. Statistical rethinking: a Bayesian course with examples in R and Stan. Boca Raton (FL): CRC Press/Taylor & Francis Group.

Mecklinger A, Pfeifer E. 1996. Event-related potentials reveal topographical and temporal distinct neuronal activation patterns for spatial and object working memory. Cognitive Brain Res. 4: 211–224.

Monfort V, Pouthas V. 2003. Effects of working memory demands on frontal slow waves in time-interval reproduction tasks in humans. Neurosci Lett. 343: 195–199.

Mudrik L, Lamy D, Deouell LY. 2010. ERP evidence for context congruity effects during simultaneous object–scene processing. Neuropsychologia. 48: 507–517.

Nieuwenhuis S, Yeung N, van den Wildenberg W, Ridderinkhof KR. 2003. Electrophysiological correlates of anterior cingulate function in a go/no-go task: Effects of response conflict and trial type frequency. Cogn Affect Behav Ne. 3: 17–26.

Nigam A, Hoffman JE, Simons, RF. 1992. N400 to Semantically Anomalous Pictures and Words. J Cognitive Neurosci. 4: 15–22.

Olofsson JK, Hurley RS, Bowman NE, Bao X, Mesulam MM, Gottfried JA. 2014. A Designated Odor-Language Integration System in the Human Brain. J Neurosci. 34(45): 14864–14873.

Olofsson JK. 2014. Time to smell: a cascade model of human olfactory perception based on response-time (RT) measurement. Front Psychol. 5.

Olofsson JK, Bowman NE, Gottfried JA. 2013. High and low roads to odor valence? A choice response-time study. J Exp Psychol Human. 39: 1205–1211.

Oostenveld R, Fries P, Maris E, Schoffelen JM. 2011. FieldTrip: Open Source Software for Advanced Analysis of MEG, EEG, and Invasive Electrophysiological Data.

Pause BM, Sojka B, Krauel K, Ferstl R. 1996. The nature of the late positive complex within the olfactory event-related potential (OERP). Psychophysiology. 33: 376–384.

Picton TW. 1992. The P300 Wave of the Human Event-Related Potential. J Clin Neurophysiol. 9: 456–479.

Polich J. 2007. Updating P300: An integrative theory of P3a and P3b. Clin Neurophysiol. 118: 2128–2148.

Polich J. 2011. Neuropsychology of P300. In: Kappenman ES, Luck SJ, editors. The Oxford Handbook of Event-Related Potential Components. Oxford (UK): Oxford University Press.

Puce A, Epling JA, Thompson JC, Carrick OK. 2007. Neural responses elicited to face motion and vocalization pairings. Neuropsychologia. 45: 93–106.

Qiu J, Luo Y, Wang Q, Zhang F, Zhang Q. 2006. Brain mechanism of Stroop interference effect in Chinese characters. Brain Res. 1072: 186–193.

Robinson AK, Reinhard J, and Mattingley JB. 2015. Olfaction Modulates Early Neural Responses to Matching Visual Objects. J Cognitive Neurosci. 27: 832–841.

Romero R, Polich J. 1996. P3(00) habituation from auditory and visual stimuli. Physiol Behav. 59: 517–522.

Ruchkin DS, Berndt RS, Johnson R, Ritter W, Grafman J, Canoune HL. 1997. Modality-specific processing streams in verbal working memory: evidence from spatio-temporal patterns of brain activity. Cognitive Brain Res. 6: 95–113.

Ruchkin DS, Johnson R, Canoune H, Ritter W. 1990. Short-term memory storage and retention: an event-related brain potential study. Electroen Clin Neurophysiol. 76: 419–439.

Sarfarazi M. 1999. Visual Event Related Potentials Modulated by Contextually Relevant and Irrelevant Olfactory Primes. Chem Senses. 24: 145–154.

Sassenhagen J, Draschkow D. 2019. Cluster-based permutation tests of MEG/EEG data do not establish significance of effect latency or location. Psychophysiology. 56: e13335.

Stan Development Team. 2017. Stan Modeling Language: User's Guide and Reference Manual (Version 2.17.0). Retrieved from mc-stan.org.

Stan Development Team. 2018. RStan: the R interface to Stan (Version R package version 2.17.3). Retrieved from http://mc-stan.org/.

Strachan RW, van Dijk HK. 2005. Weakly informative priors and well behaved Bayes factors (No. EI 2005-40). Retrieved from Erasmus University Rotterdam, Erasmus School of Economics (ESE), Econometric Institute website: https://ideas.repec.org/p/ems/eureir/7027.html

Sutton S, Braren M, Zubin J, John ER. 1965. Evoked-potential correlates of stimulus uncertainty. Science. 150: 1187–1188.

Verleger R. 1988. Event-related potentials and cognition: A critique of the context updating hypothesis and an alternative interpretation of P3. Behav Brain Sci. 11: 343–356.

Verleger R. 1997. On the utility of P3 latency as an index of mental chronometry. Psychophysiology. 34: 131–156.

Verleger R, Jaśkowski P, Wascher E. 2005. Evidence for an Integrative Role of P3b in Linking Reaction to Perception. J Psychophysiol. 19: 165–181.

Wagenmakers EJ, Marsman M, Jamil T, Ly A, Verhagen J, Love J, Selker R, Gronau QF, Šmíra M, Epskamp S, Matzke D, Rouder JN, Morey, RD. 2018. Bayesian inference for psychology. Part I: Theoretical advantages and practical ramifications. Psychon B Rev. 25: 35–57.

Xiao X, Dupuis-Roy N, Yang XL, Qiu JF, Zhang QL. 2014. The taste-visual cross-modal Stroop effect: An event-related brain potential study. Neuroscience. 263: 250–256.
